# General Suspended Printing Strategy toward Programmatically Spatial Kevlar Aerogels

**DOI:** 10.1021/acsnano.2c00720

**Published:** 2022-03-01

**Authors:** Qingqing Cheng, Zhizhi Sheng, Yongfeng Wang, Jing Lyu, Xuetong Zhang

**Affiliations:** † School of Nano-Tech and Nano-Bionics, University of Science and Technology of China, Hefei 230026, P. R. China; ‡ Suzhou Institute of Nano-Tech and Nano-Bionics, Chinese Academy of Sciences, Suzhou 215123, P. R. China; § Division of Surgery & Interventional Science, University College London, London NW3 2PF, United Kingdom

**Keywords:** aerogel, Kevlar, microgel matrix, suspended printing, thermal insulation

## Abstract

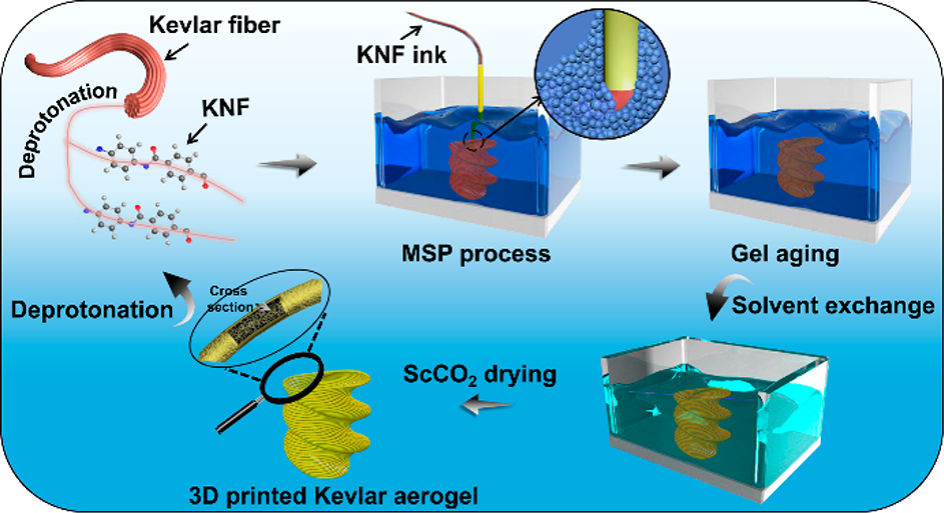

Aerogels represent a kind of nanoporous solid with immense importance for a plethora of diverse applications. However, on-demand conformal shaping capacity remains extremely challenging due to the strength unfavorable during aerogel processing. Herein, a universal microgel-directed suspended printing (MSP) strategy is developed for fabricating various mesoporous aerogels with spatially stereoscopic structures on-demand. As a proof-of-concept demonstration, through the rational design of the used microgel matrix and favorable printing of the Kevlar nanofiber inks, the Kevlar aerogels with arbitrary spatial structure have been fabricated, demonstrating excellent printability and programmability under a high-speed printing mode (up to 167 mm s^–1^). Furthermore, the custom-tailored Kevlar aerogel insulator possessing superior thermal insulation attribute has ensured normal discharge capacity of the drone even under a harsh environment (−30 °C). Finally, various types of spatial 3D aerogel architectures, including organic (cellulose, alginate, chitosan), inorganic (graphene, MXene, silica), and inorganic–organic (graphene/cellulose, MXene/alginate, silica/chitosan) hybrid aerogels, have been successfully fabricated, suggesting the universality of the MSP strategy. The strategy reported here proposes an alternative for the development of various customized aerogels and stimulates the inspiration to truly arbitrary architectures for wider applications.

Along with social development, lightweight materials are one of the hallmarks of modern society.^1−3^ The lightest material available today is regarded as the aerogel, which is defined as a three-dimensional (3D) solid interconnected network with gas-filled pores.^4−6^ Characterized by the extremely large specific area, ultralow density, and high porosity,^7,8^ aerogels manifest tremendous applications ranging from thermal/acoustic insulation of automotive and aerospace components, environmental treatments, to fabrication of energy storage components and medical devices,^9−12^ etc. Despite the outstanding potentiality, one of the major problems associated with aerogels is their on-demand conformal shaping capability.^13−17^ The application scenarios of aerogels, either as a functional component of a device or as an individual object, often have irregular appearance, so the aerogel architectures not only need to exhibit extraordinary functionalities but also require the conformal appearances with arbitrary shapes in diverse applications, such as thermal insulation of special shaped devices in aerospace vehicles and civil buildings. Therefore, the manufacturing process of application-specific conformal shaping of aerogels with a self-supporting architecture is highly demanded to further advance the application of aerogels.

Indeed, different strategies have been extensively explored for the shaping of aerogel architectures, including varieties of spatial dimensionality, i.e., spheres, fibers, films, bulks.^18−22^ For instance, aerogel microspheres have been prepared by the emulsion process and spray drying in sequence.^18^ Aerogel fibers have been produced by freeze/wet spinning,^20,23^ reaction spinning,^24^ or capillary tube-assisted sol–gel confined transition,^2^ respectively. Aerogel films have been fabricated by blade/spin/dip coating^21,25^ or drop casting.^26^ Bulk aerogels have been manufactured by vacuum filtration^22^ or freeze-drying directly.^27^ However, aerogels obtained through these traditional methods present mass production capability but limited on-demand conformal capability. Besides, although aerogels have an exceptionally high specific strength, they are generally brittle and difficult to machine by subtractive postprocessing. The sacrificial mold casting method have been applied to fabricate aerogels with complex shapes by pouring the precursor sols into the reserved cavity of the mold. After subsequent sol–gel transition, the gel can be taken out from the mold, followed by special drying.^17,28,29^ However, the resulting aerogels via this process are heavily dependent on the molds, presenting multiple steps, less shape complexity, and huge amounts of material consumption. Besides, when preparing aerogel with a sophisticated shape, the difficulty of taking the gel out from the mold may be encountered. Consequently, the fabrication approach to construct the aerogel architectures with designable and complex structures still remains challenging.

Recently, 3D printing techniques have offered enormous opportunities for customizing aerogels on the basis of their delicate design due to their modifiability and flexibility, which were generally unachievable via conventional manufacturing pathways.^30−32^ As a powerful complement to traditional shaping methods, 3D printing does not need to remove superfluous parts, thus protecting the aerogel objects from disrupting the original structure. As for the spatial dimensionality, 3D printing could prepare aerogel microspheres with excellent monodispersity and repeatability,^33^ aerogel fibers or films with customized patterns,^34,35^ and aerogel bulks with sophisticated structures.^30,31^ Currently, three major categories of 3D printing techniques have been proposed: inkjet-based printing, laser-based printing, and extrusion-based printing. Among the existing 3D printing methods, extrusion-based direct ink writing (DIW) is the most attractive candidate for constructing 3D aerogels owing to its maximum compatibility with diverse ink materials and nozzles.^14,36,37^ For example, Zhao et al. originally shifted the paradigm of a pure miniaturized silica aerogel creation away from relying on the conventional process by a DIW protocol with a slurry ink of silica aerogel dispersed in the silica nanoparticle suspension.^13^ Gao et al. printed a highly stretchable neat carbon aerogel with the aid of a DIW process due to its superiority of custom-tailored production of complex designs.^38^ Our group has also developed an improved DIW method by integrating with freeze-casting, leveraging the exceptional energy absorption property and ultralow density of Kevlar aerogels.^31^ Unfortunately, the key challenges on the 3D printing of aerogels remain elusive: First, the achievement of aerogel architectures with an arbitrary design, e.g., spatially defined feature, was limited due to the harsh 45° rule. According to the principle of gravity, an object is likely to fall if one of its suspended surfaces is at an angle greater than 45° from the vertical line.^39^ In the process of extrusion-based DIW, the adhesion between adjacent layers before complete gelling is not enough to overcome its own gravity, leading to the collapse of the structure and failure of printing. Second, improving the printing speed improves the production efficiency, but the printing speed of the DIW is usually less than 20 mm s^–1^, which is unacceptable for industrial-scale production. Finally, the stringent requirements of DIW to rheological properties of ink limit the variety of printable materials. Therefore, it is urgent to develop a general 3D printing strategy to solve the above problems and realize the on-demand integrated construction of aerogel architectures with a spatially complex 3D structure, so as to meet its rapid application, including thermal insulation, shock absorption, noise reduction, or electromagnetic shielding in certain special-shaped structure scenes, such as those encountered by spacecraft.

Herein, we develop a universal microgel-directed suspended printing (MSP) strategy for the preparation of 3D mesoporous aerogels with arbitrary spatial structures, where liquid inks are printed into a microgel matrix used for supporting the printed filaments provisionally. We adopt deprotonated Kevlar nanofiber (KNF) dispersion as the proof-of-concept demonstration to illustrate the capability of this MSP strategy, because of its exceptional mechanical attribute, chemical and thermal stability, and extensive performance in body armor and reinforcing filler, etc. To promote the adhesion between adjacent KNF filaments, appropriate microgel matrix regulation is carried out by adjusting the mass fraction of Carbopol in dimethyl sulfoxide (DMSO) and adding of cross-linking agent, 1,4-dibromo butane (Db). Due to the assistance of the as-chosen microgel matrix, the rheological property of ink is not a very strict requirement and the printing speed would also be largely improved. The KNF ink deposited into the above matrix is directly accompanied by partly in situ dynamic sol–gel transformation and followed by thoroughly static gel aging. After matrix removal, solvent exchange, and supercritical CO_2_ (Sc CO_2_) drying in sequence, the 3D printed Kevlar aerogel (3D-KA) composed of randomly entangled nanofibers is obtained. The custom-built 3D printed Kevlar aerogel insulator (3D-KAI) displays efficient thermal insulation performance, ensuring the normal discharge capacity of the drone even under 30 °C below zero. Finally, we verify the universality of the MSP strategy by using organic, inorganic, and inorganic–organic hybrid materials via their corresponding precursor inks. This MSP strategy thus provides an efficient method toward next-generation aerogels with a more delicate-shaped design and offers important robustness to various applications.

## Results and Discussion

### General Description on the MSP Strategy toward Programmatically Spatial Aerogels

**Scheme 1:**
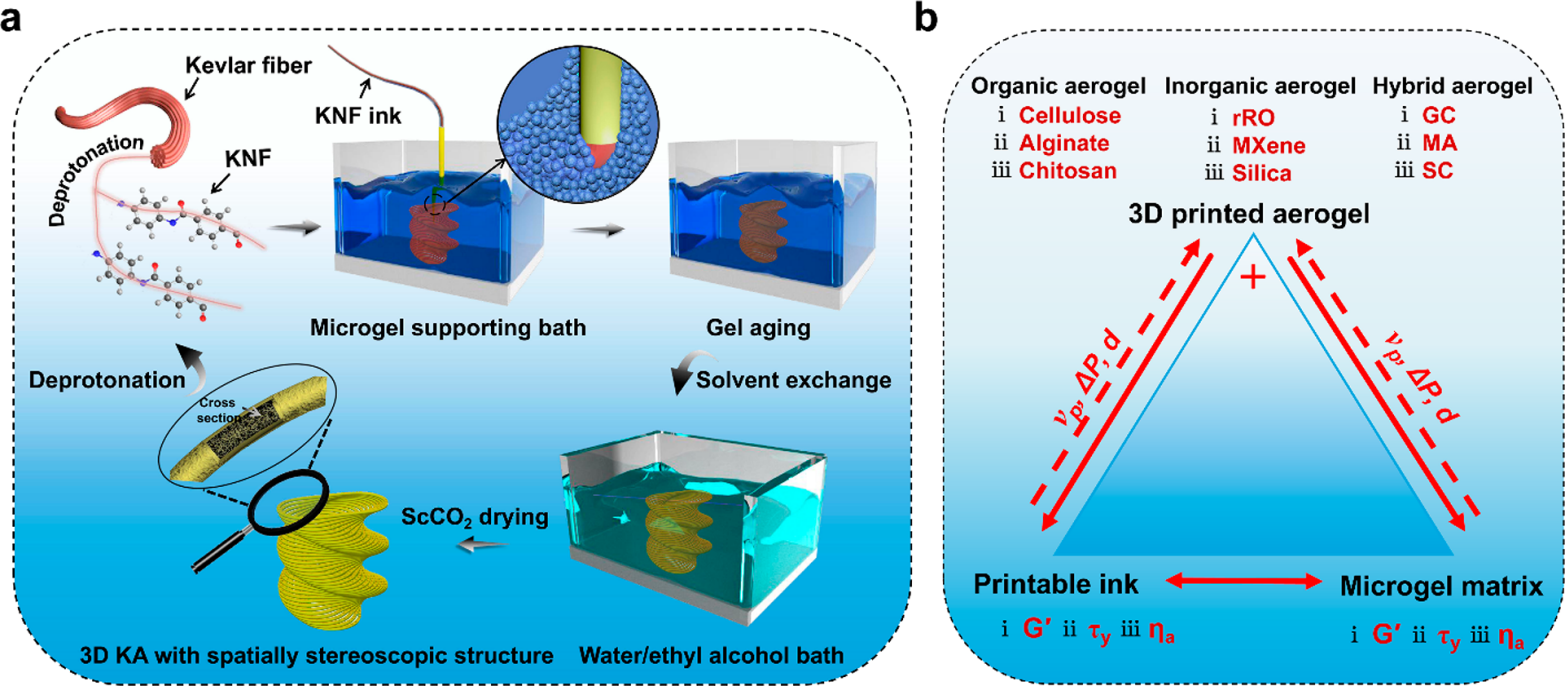
Schematics of the MSP Strategy and Parameters Associated with This Strategy: (a) Schematic Illustration of the MSP Strategy for the Preparation of 3D-KA with Spatially Stereoscopic Structure and (b) Relationship among Printable Inks, Adoptive Microgel Matrixes, and As-Printed Aerogels in the MSP Strategy) The *G*′, τ_*y*_, and η_a_ refer to storage modulus, yield stress, and apparent viscosity, respectively, and ν_p_, Δ*P*, and *d* refer to printing speed, extrusion pressure, and nozzle diameter, respectively. The rGO, GC, MA, and SC stand for reduced graphene oxide, reduced graphene oxide/cellulose, MXene/alginate, and silica/chitosan, respectively.

The MSP strategy toward 3D-KA with spatially stereoscopic structure is shown in Scheme 1a, which mainly involves the deprotonation, printing process, gel aging, solvent exchange, and Sc CO_2_ drying processes in sequence. A 2.0 wt % KNF ink was prepared by deprotonating the macroscopic Kevlar fibers in DMSO under an alkaline environment,^31,40,41^ then the MSP strategy was carried out through directly microextruding the KNF ink into a Carbopol 940-based microgel supporting bath by layer-by-layer deposition. Benefiting from the assistant effect of the microgel matrix, the deposited ink could partly form the gel in situ to protect the ink from dispersing, and the designable architecture could be supported provisionally. After thorough gelation, matrix removal, and solvent exchange in sequence, Sc CO_2_ drying was employed to obtain the final 3D-KA. It is worth mentioning that the 3D-KA can be redeprotonated into KNF ink to remold other designed architecture, exhibiting high reusability (Figure S1). The above MSP strategy could be generalized to any other kind of aerogel. The relationship among printable inks, adoptive microgel matrixes, and as-printed aerogels is demonstrated in Scheme 1b. In the MSP process, the synergistic effect between the rheological property of the printable ink and that of the microgel matrix plays a significant impact on the shaping of the resulted 3D-printed aerogel. Besides, specific ink sol–gel transition factors, e.g., pH, temperature, chemical composition, are also needed to preload into the corresponding microgel matrix to promote the shaping. For instance, storage modulus (*G*′), yielding stress (τ_*y*_), and apparent viscosity (η_a_) of the printable ink must match each other to ensure the smooth extrusion of ink from the nozzle, while appropriate *G*′, τ_*y*_, and η_a_ values of the corresponding microgel matrix are also considered to match each other to ensure stable movement of the nozzle and load-bearing of the printed filaments. In this process, the main factors affecting the morphology and diameter of the printed filament are the inner diameter of the nozzle (*d*), extrusion pressure (Δ*P*), and printing speed (ν_p_). This MSP strategy applies to various types of aerogels, i.e., organic matters (e.g., cellulose aerogel, alginate aerogel, and chitosan aerogel), inorganic matters (e.g., reduced graphene oxide (rGO) aerogel, two-dimensional carbide and nitride (MXene) aerogel and silica aerogel), and organic/inorganic hybrid matters (e.g., reduced graphene oxide/cellulose (GC) hybrid aerogel, MXene/alginate (MA) hybrid aerogel, and silica/chitosan (SC) hybrid aerogel).

### Revealing Working Mechanism of the MSP Strategy

The rheological properties of the KNF ink are demonstrated in Figure S2. The viscosity of the ink drops gradually from 10^2^ to 1 Pa s in the presence of shear flows, and the yielding stress is 120 Pa, both of which are similar to those in the previous report.^31^ Different from the traditional DIW process, where the ink has a higher storage modulus *G*′ than their loss modulus (*G*″) before the value of the yielding stress to exhibit a solidlike behavior, enabling maintained shape when the ink leaves the nozzle after being extruded, this MSP strategy does not need to undergo a rapid modulus recovery to achieve the requisite shape retention. The microgel matrix adopted here is the commercially available Carbopol power (Figure S3),^42^ which possesses tunable rheological properties over wide concentration ranges with various product categories. Owing to the rapid gelation rate of the KNF ink in protonated solvent and the significant time discrepancy of sol–gel transition between the extruded two layers,^43,44^ the surface roughness (*R*_a_) of the resulted KNF filament is only 1.46 μm (Figure S4). The smooth interface between two consecutive layers is difficult to completely merge together and a potential flaw at the merging site would occur. Consequently, we adopted a Carbopol/DMSO system to replace commonly used water to slow down the gelation rate, where the hydrogen atom in Carbopol could make ANF gel (Figure S5). Moreover, the interlayer adhesion can also be improved by adding moderate Db as the cross-linking agent^31,45^ to introduce a covalent bonding between the adjacent printed layers (Figures S6 and S7).

**Figure 1:**
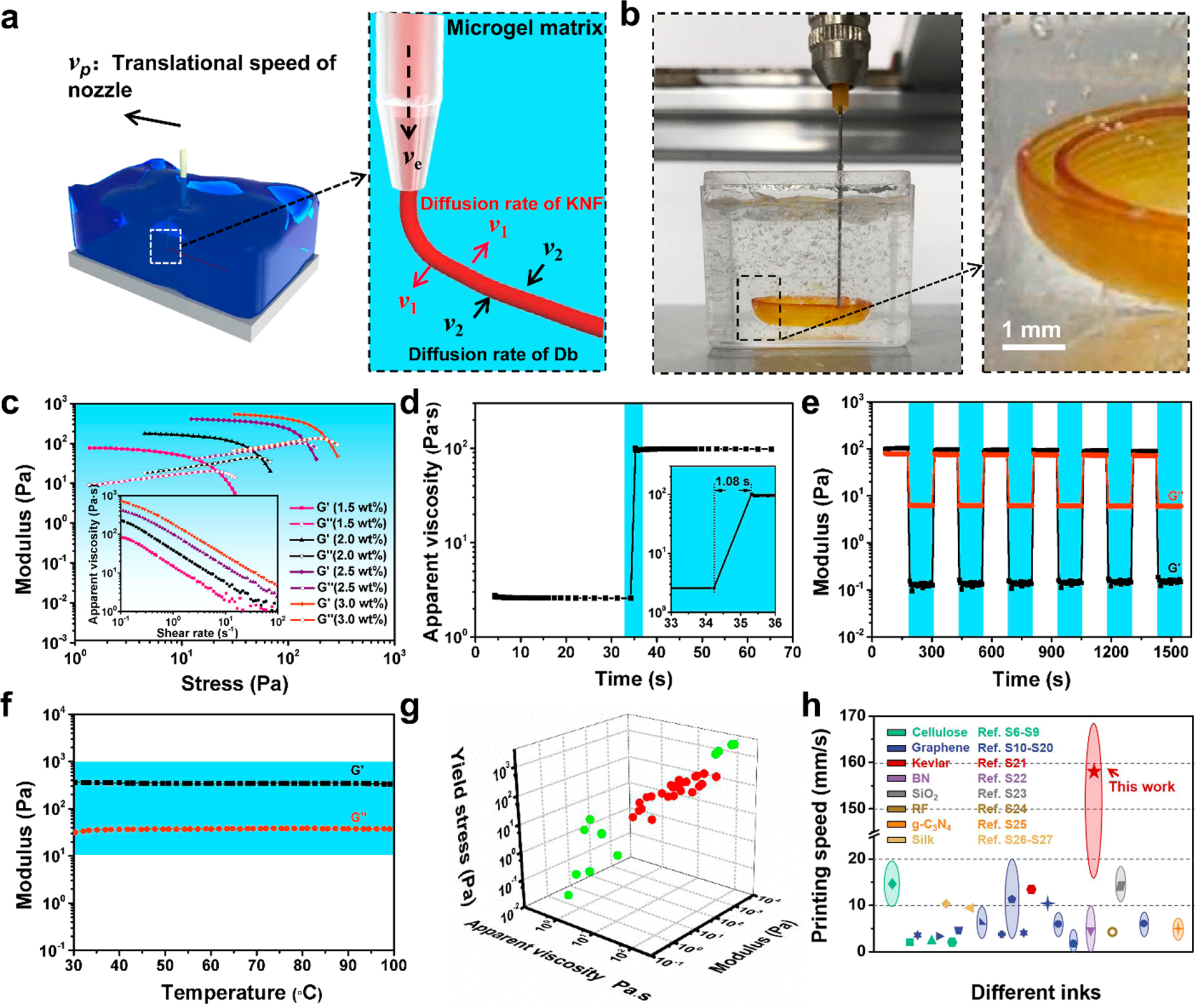
Dynamic sol–gel transition of KNF ink and rheological properties of microgel matrix in the MSP strategy. (a) The dynamic sol–gel transition process of a printed KNF filament in the microgel matrix. (b) Digital photos of the practical printing process of the KNF architecture. (c) Log–log plots of dynamic stress sweep as a function of shear stress of the microgel matrix with different percentages of Carbopol in DMSO at a constant frequency of 1 Hz. The inset in part c is log–log plots of apparent viscosity as a function of shear rate. (d) Fluidized-solidified behavior, (e) periodic oscillatory shear sweep with high-low frequency conversion, and (f) thermal stability of the optimized microgel matrix with 2.5 wt % Carbopol and 5 μL g^–1^ of 1,4-dibromo butane (Db) in DMSO. (g) Relationship between rheological parameters of the assisted microgel matrix and printability of the inks. (h) Comparison diagram of the printing speeds of reported aerogels via the extruded-based DIW process.

Figure 1a showed the dynamic printing process and dynamic sol–gel transition of the freshly printed KNF filament. Due to the external force achieved by pressurized gas and the movement of the nozzle in the supported matrix, the viscoelastic KNF ink in the syringe was extruded as an as-designed pattern readily. The diameter of filaments is decreased obviously from 253 to 96 μm with the increased printing speed from 16.67 mm s^–1^ to 166.67 mm s^–1^ and is reduced from 408 to 106 μm with decreasing nozzle diameter from 410 to 150 μm, respectively (Figures S8 and S9). In the case of nozzle diameter determination, the extrusion rate of ink (ν_e_), translational rate of the nozzle (printing speed, ν_p_), and the diffusion rate of KNF (ν_1_) and Db (ν_2_) in the supporting bath together affect the morphology and diameter of the KNF gel filament. According to the Ostwald–de Wale power law equation, the shear stress and shear rate of the ink satisfy the following empirical formula:^46^
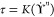
1where τ is the shear stress, ϒ̇ is the shear rate, *K* is the consistency coefficient, and *n* is the flow index. The calculated values of *K* and *n* were 12.6 and 0.79, respectively, from Figure S2a.

On the basis of fluid mechanics, the flow equation of fluid in the nozzle simplified as follows:^47^
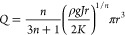
2
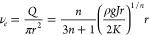
3where *Q* is the flow quantity, ρ is the fluid density, *g* is the gravitational acceleration, *r* is the nozzle inner radius, and the hydraulic slope *J* is defined as (Δ*P* is the pressure applied at the nozzle, *l* is the nozzle length). Thus, the ν_e_ is determined to be 1.09 mm s^–1^ (Δ*P* = 0.5 MPa, ρ = 1 kg m^–3^, *d* = 330 × 10^–6^ m, *l* = 38 × 10^–3^ m, and *g* = 9.8 m s^–2^). The nozzle draft ratio *i* is defined as^48^
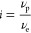
4The maximum value is 152.9 (when ν_p_ is 166.67 mm s^–1^) and mainly determined the diameter of the printed filament. In addition, the diffusion length during the dynamic sol–gel transition, *L*, was estimated using Fick’s law:^49^
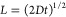
5where *D* is the diffusion coefficient and *t* is the diffusion time. Here, *D* is defined^50^ as *k*_B_*T*/(6πμ*R*) for the molecules or macromolecules in viscous fluids according to the Stokes–Einstein equation, where *k*_B_ is the Boltzmann constant, μ is the solvent viscosity, *R* is the radius of solute, and *T* is the absolute temperature. The ratio of ν_1_ to ν_2_ according to the above formula is as follows:
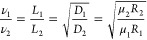
6where the subscript number of 1 stands for the diffusion of KNF into the microgel matrix and 2 stands for the diffusion of Db into KNF dispersion. The similar viscosities of the microgel matrix and KNF dispersion while the smaller molecular weights of Db (Mw is 215.91) than that of KNF (Mw is 167443) dispersion^31^ enable one to possess a similar μ_1_ value and μ_2_ value but an extremely higher *R*_1_ value than *R*_2_ value. The approximate value of , thus once printed into the microgel matrix, the diffusion of Db in the matrix would take place immediately and adjacent KNF could be successfully cross-linked, followed by a complete gel of Carbopol. Clearly, the dispersion-protonated gel achieved with DMSO and Carbopol, combined with the chemical cross-linking arising from Db, makes these three constituents suitable ingredients to tune the structural integrity of the printed KNF.

The practical printing process through a layer-by-layer building mode is demonstrated in Figure 1b and Movie S1, exhibiting the excellent printability and shaping of KNF ink. Subsequently, we investigated detailed formulation principles of the microgel matrix. Figure 1c and Figure S10 show the effect of the different percentages of Carbopol and Db on the rheological properties of the microgel matrix, respectively. All matrixes exhibit shear-thinning behavior, and 2.5 wt % Carbopol in DMSO shows a viscosity of 3–400 Pa s with the decreased shear rate from 10^2^ to 10^–1^ s^–1^, storage modulus of 400 Pa, and yield stress of 140 Pa, which is sufficient to resist against the deformation after the 3D architecture forms. The addition of Db shows a slight impact on the rheological properties of the matrix. The MSP strategy needs a rapid microgel matrix transition between the liquid and solid states, that is, when tracing out a printed path through a nozzle, the microgel fluidizes at the nozzle point and then rapidly solidifies after the nozzle shear force disappears, trapping injected ink in place.^51^ As identified at the narrow region in Figure 1d, the optimized matrix with 2.5 wt % Carbopol and 5 μL g^–1^ of Db exhibits a response time of 1.08 s, which is long enough for the rapid recovery of rheological properties. Moreover, the perfect modulus recovery of the above matrixes under periodic high-low frequency conversion displays superior stability of rheological properties of the assisted matrix (Figure 1e). In addition, the optimized microgel matrix shows excellent thermal stability over a wide range of temperatures from 30 °C to 100 °C, demonstrating versatile application even at a high temperature (Figure 1f). We further characterize the rheological properties of other ∼50 types of microgel and find that only the microgels with the rheological parameters marked in the red possess the capacity to print the KNF in practice successfully, identifying that the rheological parameters (e.g., *G*′, τ_*y*_, η_a_) of the microgel matrix are critical to achieving the suspended aerogel architectures (Figure 1g). In contrast to general DIW-based 3D printing of aerogel, whose ink adopts strict rheological properties including relatively high *G*′ and τ_*y*_ for the shape retention and self-supporting of the as-printed structure, this MSP strategy has a significant advantage on printing speed up to 166.67 mm s^–1^ (Figure 1h) and tolerant ink rheological properties (η_a_, τ_*y*_) (Table S1), which can broaden the categories of inks and improve the productivity tremendously.

### Applying the MSP Strategy into Printing of KNF Inks

**Figure 2:**
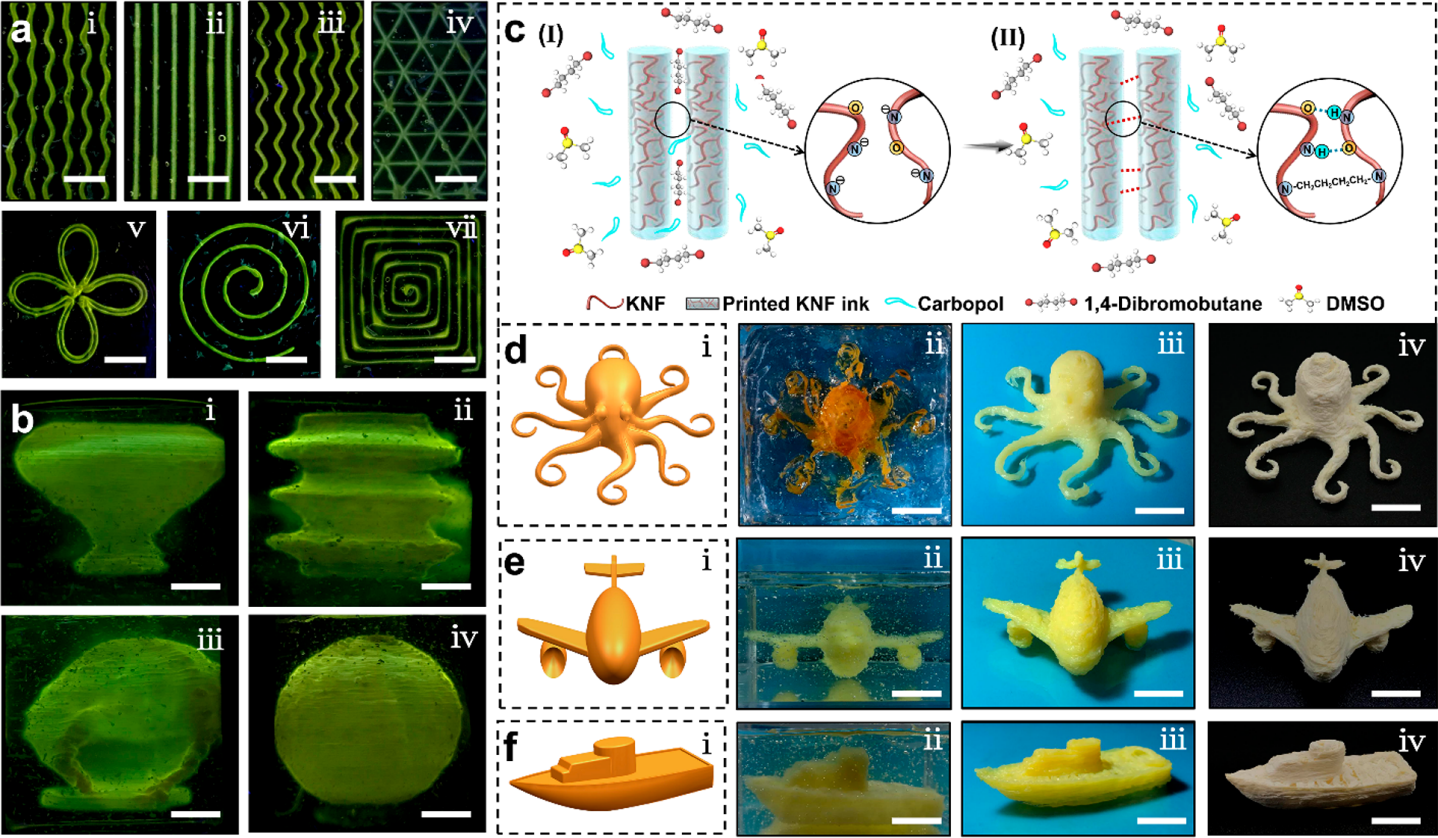
Printability and programmability of the KNF ink via the MSP strategy. (a) Digital photos of wavy (i), straight (ii), zigzag (iii), and triangular (iv) lines and complex geometries of four-leaf clover (v), Archimedean spiral (vi), and palindrome (vii) labeled with Rhodamine 6G. The scale bar is 1 mm. (b) Digital photos of shell structures of the upside-down conical flask (i), flexible tube (ii), trumpet shell (iii), and hollow sphere (iv) labeled with Rhodamine 6G. The scale bar is 1 cm. (c) Schematic illustration of the protonation and cross-linking process of two contiguous printed KNF ink in the microgel matrix. (d–f) Designed models (i), printed KNFs in microgel matrix (ii), printed KNF gels (iii), and printed KNF aerogels (iv) of the corresponding octopus, aircraft, and vessel architectures. The scale bar is 1 cm.

To explore the capability of printing KNF inks via MSP strategy, we printed varieties of different patterns, such as wavy lines, straight lines, zigzag lines, and triangular structures, and more complex geometries of four-leaf clover, Archimedean spiral, palindrome marked with Rhodamine 6G on the basis of the predesigned shapes (Figure 2a). In addition, shell structures as an upside-down conical flask, flexible tube, trumpet shell, and hollow sphere were also been printed successfully, all of which exhibited outstanding shaping and programmability (Figure 2b and Figure S11). It could be seen that any arbitrary shapes could be obtained with the assistance of the microgel matrix, promising a broad design space. For taking out 3D structures from the microgel matrix, Db was introduced as a remedy between adjacent KNF polyanion filaments to enable substitution reaction to the formation of covalent bond, which could enhance the overall structural cohesion and improve interlayer strength (Figures S12 and S13). Figure 2c demonstrates the mechanism of the deprotonated KNF printed into a microgel matrix containing DMSO, Carbopol, and Db. Carbopol is the proton donor, which protonates polyanion to form a gel, while Db is the cross-linking agent, forming covalent alkane bonds at the negative ion site. The protonation and the formation of the covalent alkane bonds are competing. Owing to both of these effects, the 3D gel objects (e.g., octopus, aircraft, and vessel) could be removed from the microgel matrix by washing with 0.1 M NaCl aqueous solution 3–5 times after static sol–gel transition thoroughly for 12 h (Figure 2d–f and Figure S14). Subsequently, after the solvent exchange with deionized water and ethanol in sequence followed by Sc CO_2_ drying, the 3D-KA with specific shapes was obtained. The resulted aerogel exhibits superior structural integrity and ultralow density of a single filament down to 36.5 mg cm^–3^. Consequently, the MSP strategy appears to be a perfect strategy to create aerogels with truly arbitrary design, and flexibility in shapes would also greatly promote the favorable use of Kevlar in a myriad of industry fields.

### Performance of the Printed Kevlar Aerogels with Spatial Architectures

**Figure 3:**
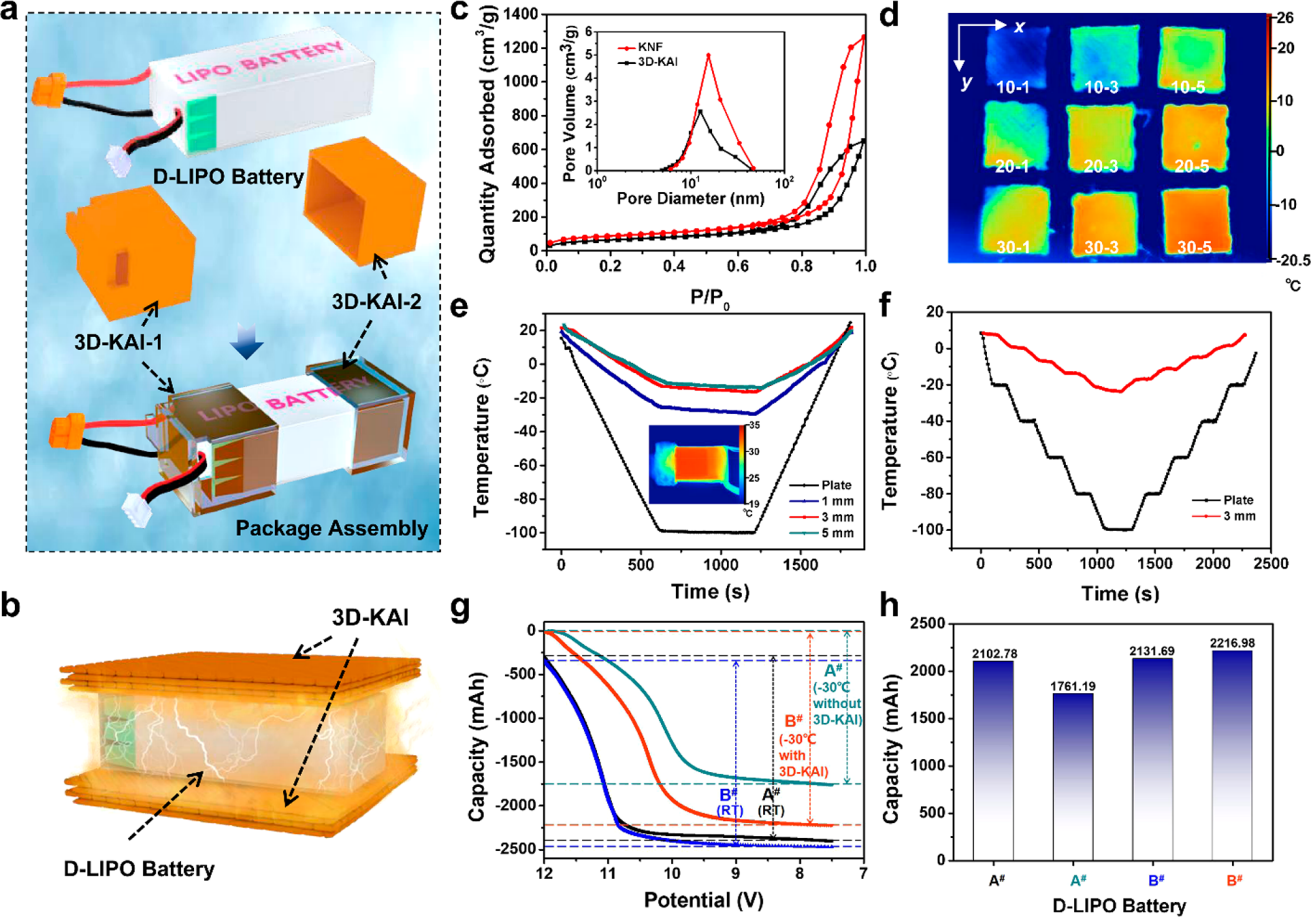
Thermal insulation performance of the 3D-KAI with spatially stereoscopic structure. (a) Designed models and package assembly of the 3D-KAI-1 and 3D-KAI-2. (b) Schematic illustration of the aerogel layer for thermal insulation performance. (c) Nitrogen adsorption–desorption isotherms of the 3D-KAI and reported KNF aerogel. The inset in part c is the corresponding pore volume. (d) Infrared images of 3D-KAI with different printing densities (10, 20, 30 mg cm^–3^, the number before hyphen) and thicknesses (1, 3, 5 mm, the number after hyphen). (e,f) Temperature–time curves of the 3D-KAI with different thicknesses (0 (i.e., plate), 1, 3, 5 mm). The inset in part e is the thermal images of the D-LIPO coated with a 3D-KAI. (g) The discharge curves of the D-LIPO with or without the coating of the 3D-KAI. (h) The capacity comparison of the D-LIPO with or without the coating of the 3D-KAI. The A^#^ in black color refers to D-LIPO labeled with A^#^ discharged at room temperature, the B^#^ in blue color refers to D-LIPO labeled with B^#^ discharged at room temperature, the A^#^ in green color refers to D-LIPO labeled with A^#^ discharged at −30 °C, the B^#^ in red color refers to D-LIPO labeled with B^#^ coated with 3D-KAI discharged at −30 °C.

In order to demonstrate the valuable application of the MSP strategy in the real fields (Figure 3a), the predesigned spatially stereoscopic 3D-KAI-1 and 3D-KAI-2 were printed via MSP strategy and packaged together to improve the performance of drone lithium polymer package battery (D-LIPO) at low temperature. Because of the intrinsically excellent thermal insulation capability of the aerogel and the extreme temperature resistance of Kevlar, the cold current could be obstructive thus the battery could still serve even at extremely low temperatures (Figure 3b). The predesigned models and printed 3D-KAI-1 and 3D-KAI-2 are shown in Figure S15. SEM images in Figure S16a illustrate an internal porous network consisting of denser entangled nanofibers interconnected with each other than reported KNF (Figure S16b). In addition, an obvious alkyl peak appears for the 3D-KAI while an almost flat and negligible peak is observed for the KNF aerogel at 2853, 2924, and 2961 cm^–1^ (Figure S17), indicating a successful grafting of the alkyl group. The close-up micrograph shows a nearly perfect bonding at the interface of adjacent layers (Figure S16c), verifying the good cohesion in the interlayer region of two consecutive layers. Thereby, the key bottleneck of the defect in the cohesion in constructing KNF aerogel architectures can be effectively eliminated. In addition, a main decomposition process of 3D-KAI appears at 508.4–580.9 °C, while the thermal decomposition temperature of the reported KNF aerogel was 513.2–587.6 °C, indicating that the introduction of alkyl groups has little effect on the thermal stability of the printed architecture (Figure S18). Besides, the type IV nitrogen adsorption/desorption isotherm and the pore size distribution demonstrated the *S*_BET_ and pore volume of 3D-KAI are 230.2 m^2^ g^–1^ and 1.007 cm^3^ g^–1^, respectively, while those of reported KNF aerogel are 307.5 m^2^ g^–1^ and 1.955 cm^3^ g^–1^, respectively (Figure 3c); the slight deviation might be caused by the increase of cross-linking density.

The thermal imaging when placing 3D-KAI on a cold plate of −20 °C reveals that the thermal insulation properties are tightly related to the printing density and thickness of the 3D-KAI (Figure 3d). The better thermal insulation performance could be obtained by increasing the printing density and thickness, exhibiting tailorable thermal management properties arising from rationally engineered design. Appropriately choosing thermal management scenario, the shape-specific 3D-KAI with a thickness of 3 mm and an ultralow thermal conductivity of 0.0279 W m^–1^ K^–1^ could be customized (the inset in Figure 3e), the infrared image demonstrates that the 3D-KAI can effectively trap the heat for the drone lithium polymer package battery (D-LIPO) under low temperatures. The temperature–time curves provide that the surface temperature of the cold plate decreases with the injection of liquid N_2_ and reaches an equilibrium of −100 °C, while the equilibrium temperatures of 3D-KAI with a thickness of 1, 3, and 5 mm are −30, −16, and −14 °C, respectively (Figure 3e), further demonstrating that a better heat insulation performance could be obtained with the increase of the thickness. By linearly stepwise decreasing the temperature until equilibrium, the 3D-KAI with a thickness of 3 mm shows a rapid and stable thermal insulation response (Figure 3f). In continuation, more complex aerogel devices can be customized directly by rationally designing the geometries in advance. The D-LIPO coated with customized 3D-KAI assembly shows outstanding capacity retention of 2216.98 mAh even under −30 °C due to the excellent thermal insulation performance, 25.88% higher than the value of 1761.19 mAh of the D-LIPO without thermal insulators (Figure 3g,h), indicating the battery could be protected efficiently to ensure normal discharge capacity. The 3D-KAI enabled by the MSP strategy can accurately replicate the multiscale architecture of practical devices and solve the drawback of the capacity decline of the battery operating at low temperatures, which could be expanded to other thermal insulation circumstances. Therefore, the MSP strategy has been proven to be a suitable approach to prepare aerogel objects on demand for specific applications where complicated architectures are required.

### Universality Demonstration of the MSP Strategy

**Figure 4:**
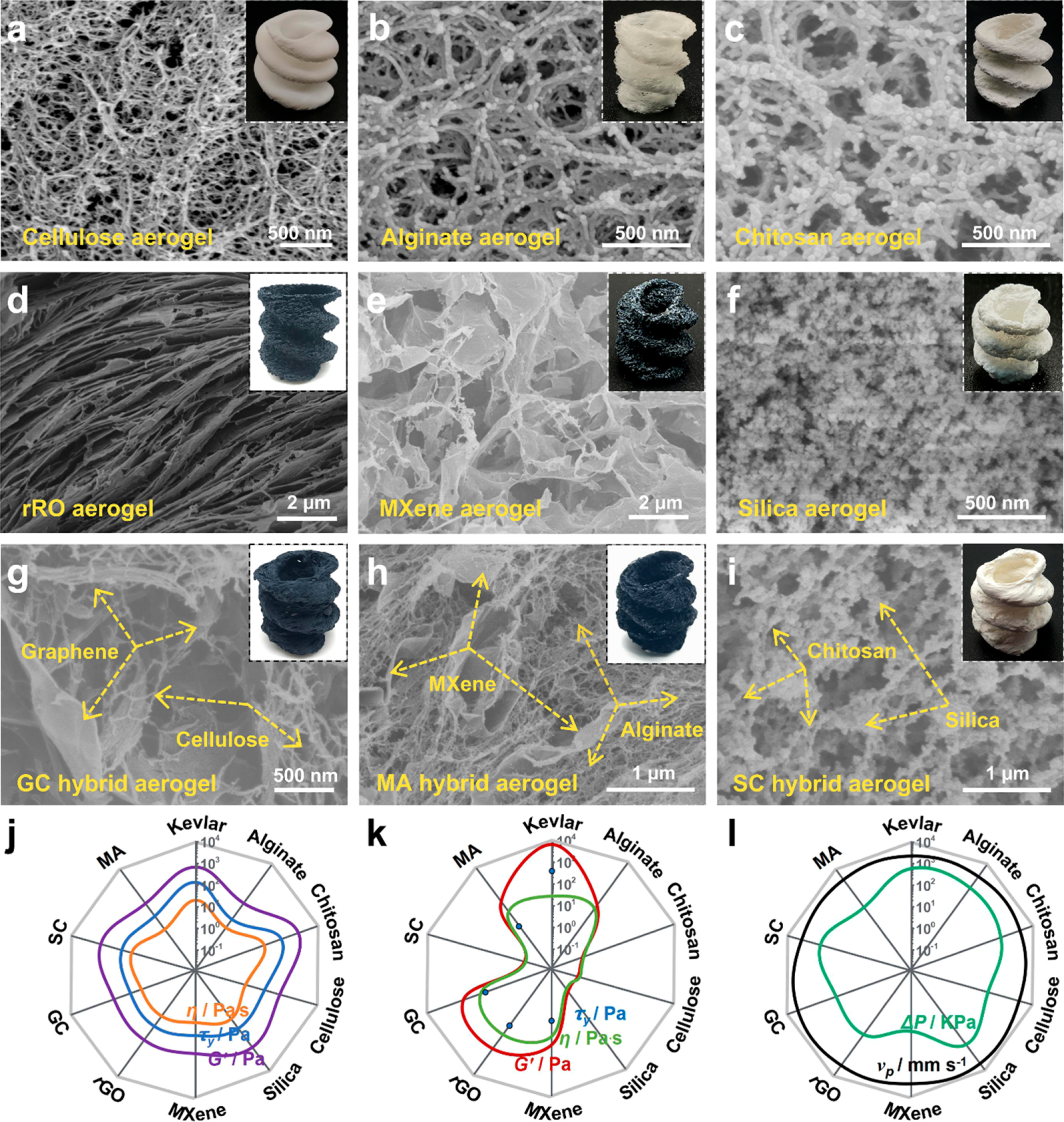
Various types of 3D printed aerogel spirals constructed via the MSP strategy: (a) cellulose, (b) alginate, (c) chitosan, (d) rGO, (e) MXene, (f) silica, (g) graphene/cellulose, (h) MXene/alginate, and (i) silica/chitosan. The rheological properties of corresponding microgel matrixes (j) and corresponding inks (k) in the MSP strategy. (l) The extrusion pressure and printing speed used in the MSP strategy.

Thousands of aerogels with various performances have been proposed so far, all of which are of crucial importance for real-life applications. In order to overcome the poor machinability of aerogels caused by the poor mechanical strength, various improved DIW techniques were also born to prepare aerogels with various shapes and properties. However, these shapes were generally limited to the quasi-two-dimensional plane structure, and the manufacturing of functional single component and heterogeneous materials with spatially arbitrary and exquisite features was unavailable. The MSP strategy with excellent shaping capabilities provides a way toward the rapid prototyping of 3D aerogel objects with the assistance of the alternative microgel matrix. In order to verify the universality of this strategy, organic matter (cellulose, chitosan, and alginate), inorganic matter (rGO, MXene, and silica), and inorganic/organic hybrid matter (rGO/cellulose (GC), MXene/alginate (MA), and silica/chitosan (SC)) aerogels were printed successfully via corresponding precursor inks (Movies S2–S4). As demonstrated in Figure 4, all the 3D-printed aerogel objects show an almost perfect spiral shape predesigned by the computer-aided design (CAD) and a porous structure in the cross-section, indicating the superior shaping of aerogel precursors and the universal applicability of the MSP strategy. In addition, the morphology of the above-mentioned 3D aerogel objects with dissimilar aggregated structures was similar to those of the respective bulk aerogels reported before,^10,24,52−55^ indicating that the MSP strategy would not influence the microstructure of the 3D printed aerogel objects. For instance, organic aerogels prepared from the nanofiber-based precursor, e.g., cellulose nanofiber, chitosan nanofiber, and alginate nanofiber, exhibited as collectives of polymeric nanofibers with different diameters (Figure 4a–c), while rGO aerogel and MXene aerogel exhibited an obvious 3D network consisting of overlapping sheets (Figure 4d,e). The resulted aerogels would inherit the properties and functions from the parent building blocks while maintaining the aerogel nature. The exploitation of various aerogels with particular structures has emerged as a potent platform to accelerate materials innovation. For example, as a fascinating nature of biocompatibility, a cellulose nanofiber-based spatially stereoscopic aerogel may offer more possibilities in next-generation biomedical devices for cell culturing or biosensors, etc. It was worth mentioning that the pure silica aerogel characterized as fragile was originally printed into the spatially stereoscopic structure without the addition of any other component, breaking the limitation of extruded-based 3D printing to the ink’s viscoelastic properties (Figure 4f). Because the specific elastic bending strain of the ceramic is known to be intrinsically inferior to that of polymer or carbon, it would be quite challenging to achieve. The resulting silica aerogel is comprised of the featured pearl necklace-like structure, which is similar to the previous report.^24^ In addition, the hybrid aerogels composed of organic matter and inorganic matter also demonstrate the flexibility of the MSP strategy (Figure 4g–i). On the other hand, the highly specific surface area and large pore volume of all kinds of 3D printed aerogel objects also proved the success in printing the aerogel objects via the MSP strategy (Figures S19 and S20). The rheological properties of the above used microgel matrixes and inks shown in Figure 4j,k, where the *G*′, τ_*y*_, and η_a_ have the same trend. The used Δ*P* and printing speed were demonstrated in Figure 4l, where the speed could also be improved by regulating appropriately the Δ*P* and the rheological properties of microgel matrixes and corresponding inks. Consequently, MSP strategy has been proven as an even more powerful manufacturing asset that not only adds on an extra dimension for aerogels in the available design space, but also is likely to integrate performance prediction with the flexible structural design to elicit desired functionality of aerogels in the future, which could be significant for widespread applications.

## Conclusions

In summary, this work offers a universal MSP strategy, i.e., microgel-directed suspended printing strategy, to enable the implementation of enticing spatially stereoscopic architectures into 3D aerogel objects. As a proof of concept, the 3D-KA with exceedingly spatial programmability was constructed by regulating the formulation of the assisted matrix. The MSP strategy powerfully breaks the limitation of current extruded-based 3D printing to the ink’s viscoelastic properties and greatly improves the printing speed. In a special scenario, the custom-built 3D-KAI provides a superior thermal insulation performance for the D-LIPO due to its hierarchically porous structure, which is favorable to ensure normal discharge capacity even at −30 °C. Finally, a wide variety of spatially programmable aerogels has been constructed by this robust strategy, which provide a universal insight into the design of aerogels and will promote their applications undoubtedly. Although it covers a much narrower range as compared with the traditional bulk aerogels, the 3D printed aerogel objects with arbitrary spatially stereoscopic architectures prepared via this versatile strategy will be a giant leap forward for the manufacturing industry.

## Materials and Methods

### Materials

Kevlar 1000D was obtained from Dongguan SOVETL Co., Ltd. Potassium hydroxide (KOH), 1,4-dibromo butane, sodium alginate and gelatin, hydroiodic acid, and Rhodamine 6G were purchased from Aladdin Co. DMSO, ethanol, tertiary butanol, glycerol, ammonia aqueous solution (NH_3_·H_2_O, 25–28 wt %), sodium chloride (NaCl), calcium chloride dihydrate (CaCl_2_·2H_2_O), formic acid, and chitosan were purchased from Sinopharm Chemical Reagent Co., Ltd. Carpobol 940 and U20 were purchased from Guangzhou Bailijia Technology Co. Ltd., and l-ascorbic acid was purchased from Macklin Co., Ltd. Cotton balls were kindly provided by Winner Medical Supplies Co., Ltd. 1-Allyl-3-methyllmldazollum chloride was purchased from Energy Chemical Co. Graphene oxide (GO), Ti_3_C_2_ MXene, and condensed silica (CS) colloidal particles were synthesized in our group; cellulose nanofiber was kindly provided by Jiangsu North Century Cellulose Material Co., Ltd. Deionized water (18.2 MΩ cm^–1^) was obtained from a Millipore-Q system. All other reagents were used without further purification.

### Fabrication of 3D Printed KNF Aerogel

The 2.0 wt % KNF dispersion ink was prepared by mechanical stirring a mixture of macroscopic Kevlar fiber (2.0 g) and DMSO (96.0 g) in the presence of KOH (2.0 g) for 1 week.^31^ A bit of Rhodamine 6G was added to observe the geometries. For the corresponding microgel matrix, Carpobol 940 (2.5 g) was dissolved into the DMSO (100 mL), and then different contents (0, 2.5, 5, 7.5 μL g^–1^) of Db was added by mechanical stirring, and the bubbles were removed by centrifugation. To obtain the 3D KNF architecture with a predesigned structure, the viscous KNF ink was loaded into the dispensing valve under constant extruding pressure of 0.5 MPa, and then the KNF ink was printed into a microgel matrix in the form of layer by layer using a 3-axis robotic deposition system. The as-printed 3D KNF stood for 12 h to ensure thorough gelation. After solvent-exchange with 0.1 M NaCl aqueous water, deionized water, and ethanol in sequence, Sc CO_2_ drying was applied to obtain the final 3D printed KNF aerogel architecture.

### Fabrication of 3D Printed Nanofibrous Cellulose Aerogel

To prepare the cellulose nanofiber dispersion ink, a cotton ball (2.0 g) was dispersed into 1-allyl-3-methyllmldazollum chloride (50 mL) at 60 °C for 12 h, and then the dispersion was diluted with DMSO until the concentration was 2.0 wt %.^26^ The corresponding assisted matrix was prepared by dissolving Carpobol 940 (1.2 g) into a mixture (100 mL) of DMSO and deionized water with a volume ratio of 4:1, followed by gelation by adding KOH (100 μL, 10 M) and removing bubbles by centrifugation. Subsequent 3D printing, gel aging, solvent exchange, and the Sc CO_2_ drying process were the same with the KNF aerogel architecture, except that the extruding pressure was 0.05–0.1 MPa, a moving speed was 42 mm s^–1^, and the diameter of the nozzle was 410 μm.

### Fabrication of 3D Printed Nanofibrous Chitosan Aerogel

Chitosan powder (5.0 g) was dissolved into formic acid aqueous solution (3.0 wt %, 100 mL) at 60 °C for 12 h to obtain the chitosan nanofiber dispersion ink. Then Carbopol U20 power (1.0 g) was dissolved into deionized water (100 mL), and then KOH (1.5 mL, 10 M) was added to make the system gelation. After removing the bubbles by centrifugation, the suspended matrix was prepared successfully. Subsequent 3D printing, gel aging, solvent exchange, and the Sc CO_2_ drying process were the same with the KNF aerogel architecture, except that the extruding pressure was 0.25–0.3 MPa, a moving speed was 40 mm s^–1^, and the diameter of the nozzle was 410 μm.

### Fabrication of 3D Printed Nanofibrous Alginate Aerogel

For the sodium alginate nanofiber dispersion ink, sodium alginate powder (4.0 g) was dissolved into deionized water (100 mL) completely at 60 °C for 6 h. For the corresponding microgel matrix, 2.0 wt % gelation was prepared by dissolving gelation power (4.5 g) into deionized water (225 mL) containing 0.1 M CaCl_2_·2H_2_O at 60 °C for 2 h and then stored at 4 °C overnight. The resulting bulk gelation was transformed into a microgel by mechanical stirring for 5 min. Subsequent 3D printing and gel aging processes were the same with the KNF aerogel architecture, except that the extruding pressure was 0.45–0.5 MPa, a moving speed was 35 mm s^–1^, and the diameter of the nozzle was 330 μm. After solvent-exchange with deionized water and ethanol in sequence, Sc CO_2_ drying was applied to obtain the final 3D printed alginate aerogel architecture.

### Fabrication of 3D Printed rGO Aerogel

The GO dispersion ink with a concentration of 15 mg mL^–1^ was prepared according to the procedure reported in our group.^10^ The preparation of the corresponding microgel matrix and 3D printing process was the same with the alginate aerogel architecture, except that the extruding pressure was 0.01–0.02 MPa and the moving speed was 45 mm s^–1^. After that, the as-printed architecture gelled thoroughly within 12 h, then 10 wt % hydroiodic acid was added into the architecture and put into an oven of 60 °C for 6 h, and then the printed architecture was washed with hot water at 60 °C and solvent exchanged with ethanol, followed by the Sc CO_2_ drying process.

### Fabrication of 3D Printed MXene Aerogel

The MXene dispersion ink with a concentration of 20 mg mL^–1^ was prepared according to the procedure reported in the literature.^55^ The preparation of the corresponding microgel matrix, 3D printing, gel aging, solvent exchange, and Sc CO_2_ drying process were the same with the alginate aerogel architecture, except that the concentration of CaCl_2_·2H_2_O was 0.2 wt %, the extruding pressure was 0.01–0.02 MPa, the moving speed was 47 mm s^–1^, and the diameter of the nozzle was 410 μm.

### Fabrication of 3D Printed Silica Aerogel

The CS ink was prepared according to the procedure reported in our group,^24^ and then Carbopol 940 power (2.0 g) was added into CS solution (100 mL) by the continuous stirring process until dissolved completely. Subsequently, Carbopol 940 power (0.5 g) was dissolved completely into deionized water (100 mL), and then NH_3_·H_2_O was added to make the system with a base concentration of 2 M. After removal of the bubbles by centrifugation, the available assisted matrix was prepared successfully. Subsequent 3D printing, gel aging, solvent exchange, and the Sc CO_2_ drying process were the same with the KNF aerogel architecture, except that the extruding pressure was 0.3 MPa, the moving speed was 56 mm s^–1^, and the diameter of the nozzle was 410 μm.

### Fabrication of 3D Printed GC Aerogel

The 5.0 wt % cellulose nanofiber dispersion was homogeneously mixed with 15 mg mL^–1^ GO aqueous solution at a mass ratio of 1:1. The preparation of the assisted matrix, 3D printing, and gel aging processes were the same with the alginate aerogel architecture, except that the concentration of CaCl_2_·2H_2_O was 0.5 wt %, the extruding pressure was 0.02 MPa, the moving speed was 52 mm s^–1^, and the diameter of the nozzle was 330 μm. The as-printed architecture gelled thoroughly within 12 h and then was soaked in l-ascorbic acid aqueous solution at 50 °C for 12 h. The subsequent solvent exchange and Sc CO_2_ drying processes were the same with the alginate aerogel architecture.

### Fabrication of 3D Printed MA Aerogel

The 4.0 wt % sodium alginate dispersion was mixed with 20 mg mL^–1^ MXene aqueous solution at a mass ratio of 1:1. The preparation of the corresponding microgel matrix, 3D printing, gel aging, solvent exchange, and Sc CO_2_ drying processes were the same with the alginate aerogel architecture, except that the extruding pressure was 0.01–0.1 MPa, the moving speed was 40 mm s^–1^, and the diameter of the nozzle was 510 μm.

### Fabrication of 3D Printed SC Aerogel

The 5.0 wt % chitosan dispersion was mixed with CS solution at a mass ratio of 2:1. Then, 0.5 wt % Carbopol U20 microgel was prepared by dissolving Carbopol U20 powder (0.5 g) into deionized water (100 mL) with a concentration of an aqueous ammonia solution of 1.0 M, and after bubble elimination by centrifugation, the assisted matrix was prepared. Subsequent 3D printing, gel aging, solvent exchange, and the Sc CO_2_ process were the same with the chitosan aerogel architecture, except that the diameter of the nozzle was 330 μm.

### Characterization

The related steady shear rate sweep and the dynamic stress sweep of the inks and microgel matrixes as well as the periodic oscillatory shear rate sweep and temperature sweep of the matrixes were determined by a rotational rheometer (RS6000, Haake, Germany) with a gap of 1 mm, and the constant frequency is 1 Hz. The fluorescence images of the printed gel models labeled with Rhodamine 6G were captured by a camera in an ultraviolet observation box (ZF-7, Qinke Analyzer, Shanghai, China). An inverted fluorescence microscope equipped with a CCD video camera (Axio Vert A1, Carl Zeiss, Germany) was used to observe and measure the diameter of the printed filaments. The morphology of the printed aerogels was characterized by scanning electron microscopy (SEM, S-4800, Hitachi, Japan) at an acceleration voltage of 5 kV. The structure of the samples was determined by the Fourier transform infrared spectroscopy (FTIR 5700, FL, USA) over 64 scans recorded with a resolution wavelength of 4 cm^–1^. The thermal stability was determined by using a thermal gravimetric analyzer (TGA, 209F1, NETZSCH, Germany) with a heating rate of 10 K min^–1^ in a nitrogen atmosphere. The pore size distribution and average pore diameter were analyzed by the Barrett–Joyner–Halenda (BJH) nitrogen adsorption and desorption method (ASAP 2020, Micromeritics, USA), and the specific surface area and pore volume were determined by the Brunauer–Emmett–Teller (BET) method, based on the amount of N_2_ adsorbed at a pressure of 0.05 < *P*/*P*_0_ < 0.3. The infrared thermal images were taken by an infrared camera (Escalab 250 Xi, Thermo Scientific, USA), and the working distance was about 30 cm. The thermal conductivity of the samples was measured by a transient hot-wire method (TC3010L, XIATECH Co., Ltd., China). The temperature of samples was monitored and recorded with thermal couples connected to a temperature controller (Hangzhou Supmea Co., Ltd., China). The low temperature was regulated by a temperature controller and control rate freezer connected to a cooling/heating stage (RTL450, Huozi Instrument Tech., Co., Ltd., China). The low-temperature discharge curves of the D-LIPO were determined by the battery test system (CE-5008–20 V10A-SMB, NEWARE, China) connected to a high-low temperature test chamber (GX-3000, GAOXIN, China). Before the low-temperature discharge test, the charged D-LIPO labeled with 1 and 2 were discharged at room temperature to ensure the same capacities. After that, the two batteries were charged fully and coated with or without 3D printed KNF insulators and then put into the high-low temperature test chamber. The discharge started when the chamber lowered the temperature from 25 °C down to −30 °C at a cooling rate of 5 °C min^–1^.

## Supporting Information Available

3D printing process of Kevlar nanofiber ink via the MSP strategy (MP4)
3D printing process of organic inks via the MSP strategy (MP4)
3D printing process of inorganic inks via the MSP strategy (MP4)
3D printing process of organic/inorganic hybrid ink via the MSP strategy (MP4)
Morphology, rheological properties, protonation and cross-linking mechanism, fluorescence micrographs and histogram, digital photos, FTIR spectra, TGA curves, nitrogen adsorption–desorption isotherms, pore volume distribution and BET surface areas, and comparison of rheological parameters of reported ink materials and the printing speeds to reported references (PDF)
