# Supplementary material for: General Suspended Printing Strategy toward Programmatically Spatial Kevlar Aerogels
Source: ACS Nano. 2022 Mar 1;16(3):4905–16. doi: 10.1021/acsnano.2c00720 (PMC9097582; doi:10.1021/acsnano.2c00720)
Supplement: Supplementary file 5 — nn2c00720_si_005.pdf [file nn2c00720_si_005.pdf]

Supporting Information

General Suspended Printing Strategy towards Programmatically Spatial Kevlar Aerogels

*Qingqing Cheng[a,b], Zhizhi Sheng[b], Yongfeng Wang[b], Jing Lyu[b], Xuetong Zhang[b,c]**

[a]School of Nano-Tech and Nano-Bionics, University of Science and Technology of China, Hefei 230026, P. R. China.

[b]Suzhou Institute of Nano-tech and Nano-bionics, Chinese Academy of Sciences, Suzhou 215123, P. R. China.

[c]Division of Surgery & Interventional Science, University College London, London NW3 2PF, United Kingdom.

*Email: xtzhang2013@sinano.ac.cn **or** xuetong.zhang@ucl.ac.uk

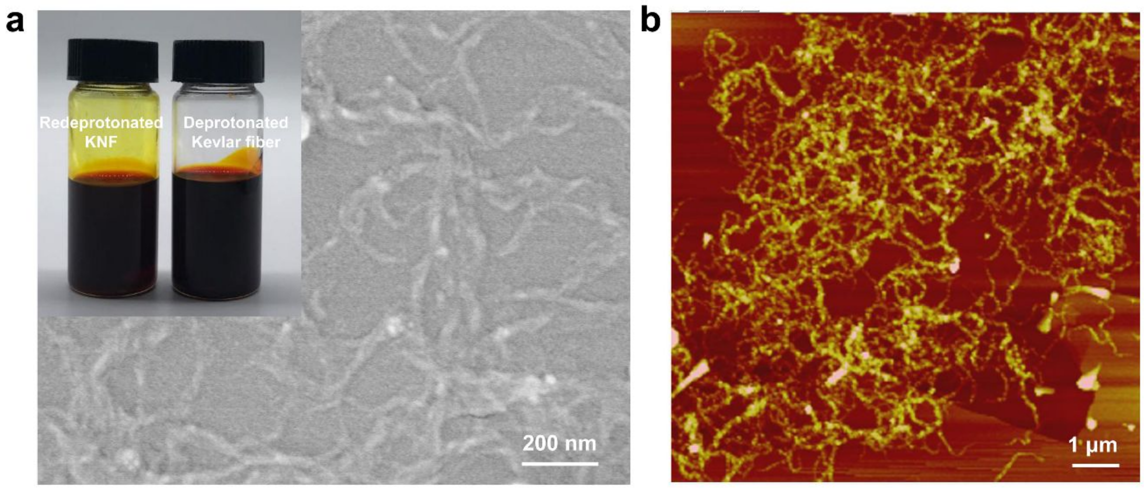

Figure S1. (a) SEM and (b) AFM images of the morphology of re-deprotonated 3D-KA. The inset in (a) was the digital photos of re-deprotonated 3D-KA and deprotonated Kevlar fiber. The 3D-KA could be re-deprotonated into KNF with the diameter of 5 - 30 nm and length of several micrometers, which was similar to the deprotonated Kevlar fiber. The re-deprotonated KNF ink could be reprinted into other designed architecture, showing the high reusability.

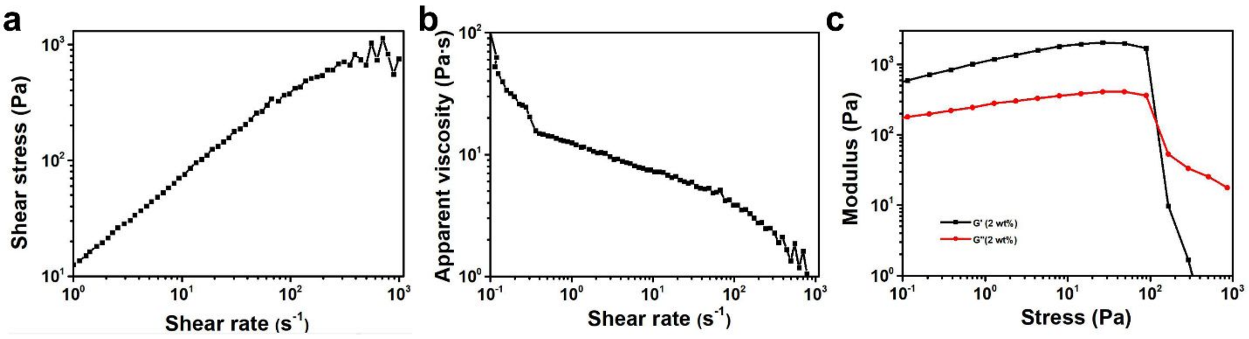

Figure S2. Rheological properties of the 2 wt% KNF ink. (a) The relationship between shear rate and shear stress. (b) Log-log plot of apparent viscosity as a function of shear rate, showing an obvious shear thinning behavior where viscosity was dependent on the desired shear rate. (c) Log-log plot of dynamic stress sweep as a function of shear stress from 10^{-1} to 10^{3} Pa at a constant frequency of 1 Hz, showing the storage and loss modulus was 2 KPa and 400 Pa respectively.

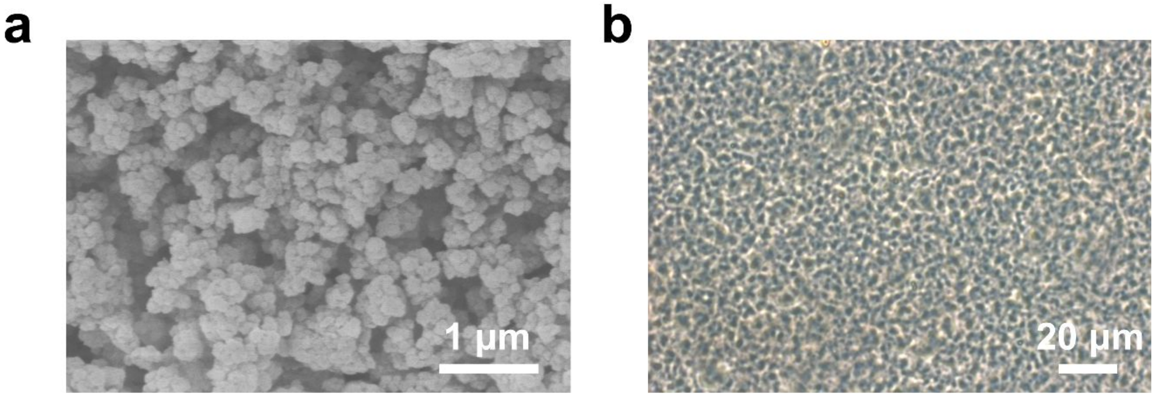

Figure S3. (a) The SEM images of the morphology of the Carbopol 940 power. (b) The fluorescence micrograph of the swollen Carbopol 940 power in DMSO. Benefiting from the powder form, the Carbopol 940 could swell into microgel, which was beneficial to use as an assisted matrix for 3D printing.

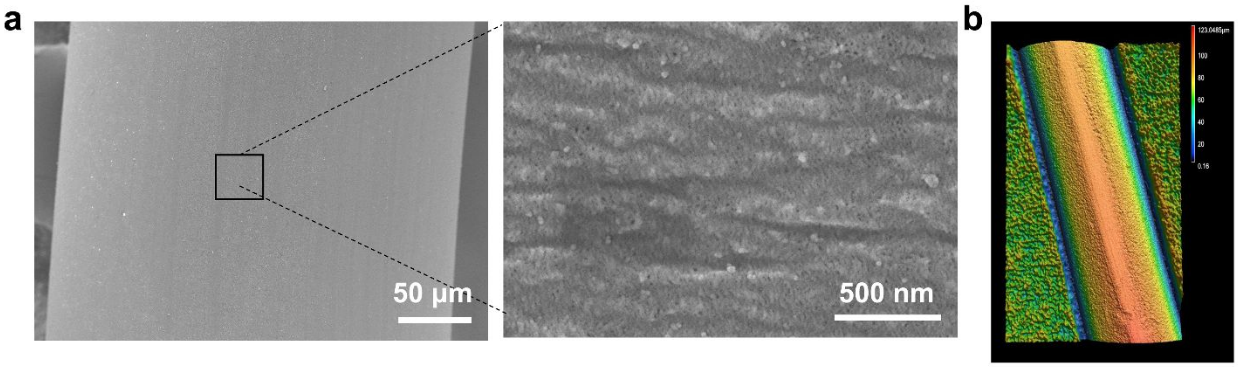

Figure S4. (a) SEM images of the surface morphology of the 3D printed KNF filament after Sc CO_2 drying when print in a water bath directly. (b) 3D confocal microscope image of the 3D printed KNF filament after Sc CO_2 drying. Due to the rapid sol-gel transition rate of the KNF ink in a protonated solvent, the arithmetical mean deviation of the profile (Ra) was 1.46 μm, which was adverse to interlayer bonding.

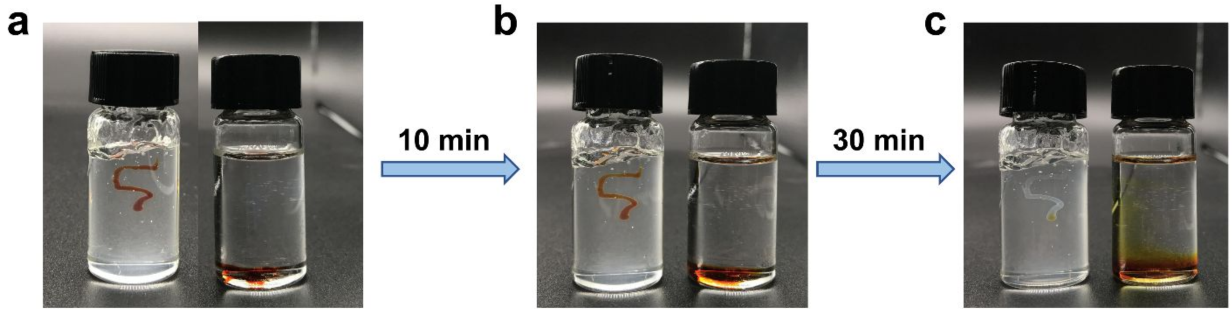

Figure S5. Digital photos of direct micro-extrusion KNF ink into different systems: 2.5% Carbopol 940 in DMSO without Db (left) and DMSO (right). When the KNF ink was extruded into the two systems, the microgel matrix could maintain the shape but dispersion in DMSO system. After 30 min, the KNF ink gradually gelled in the 2.5% Carbopol 940/DMSO microgel matrix but still dispersion in DMSO, which demonstrating that the hydrogen atom in Carbopol could make ANF gel.

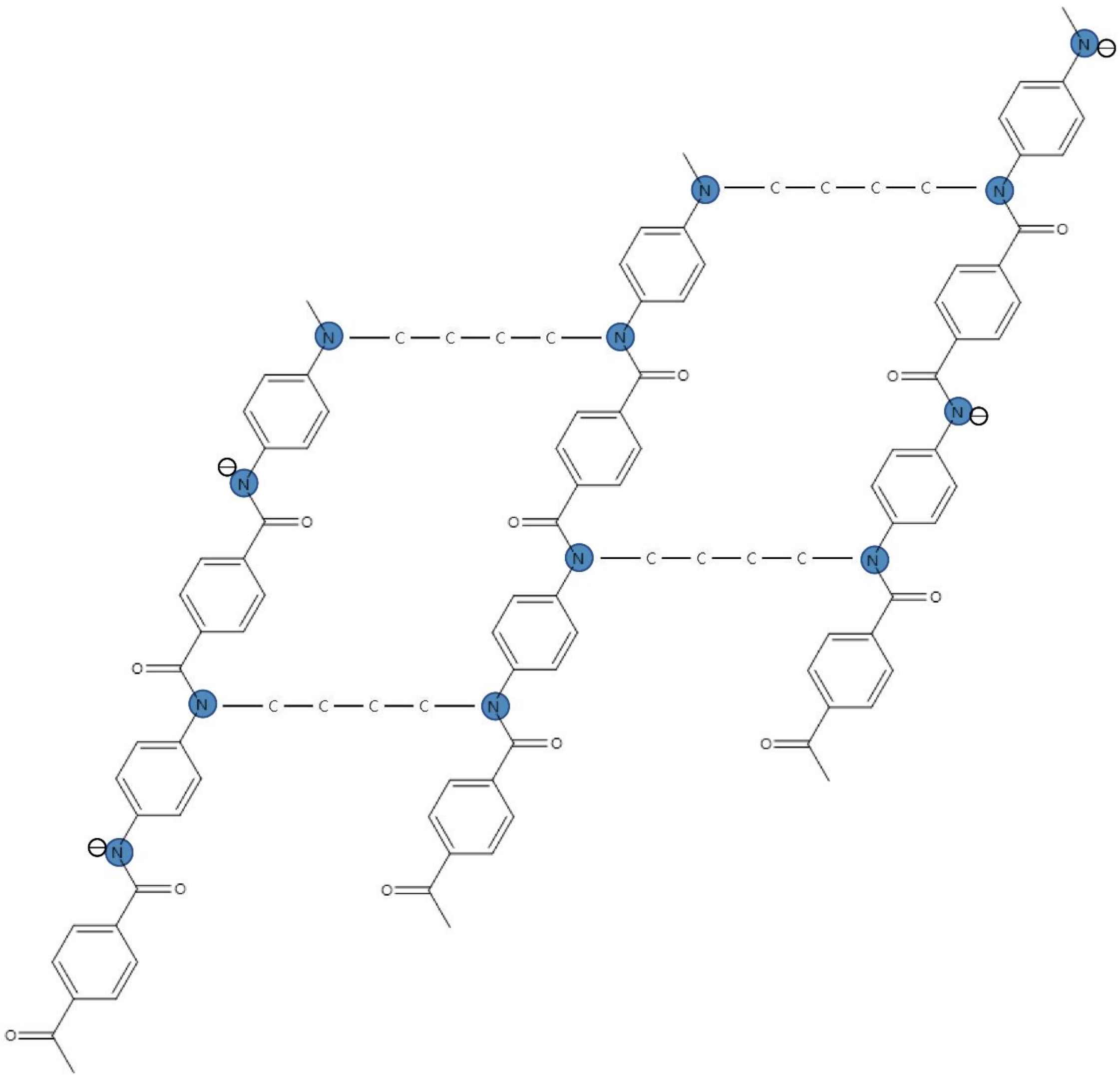

Figure S6. The chemical structures of deprotonated polyparaphenylene terephthalamide (PPTA, base constituent of Kevlar) cross-linked with Db. From the structural formula, adjacent molecular chains can be covalently crosslinked and interlayer adhesion can also be improved.

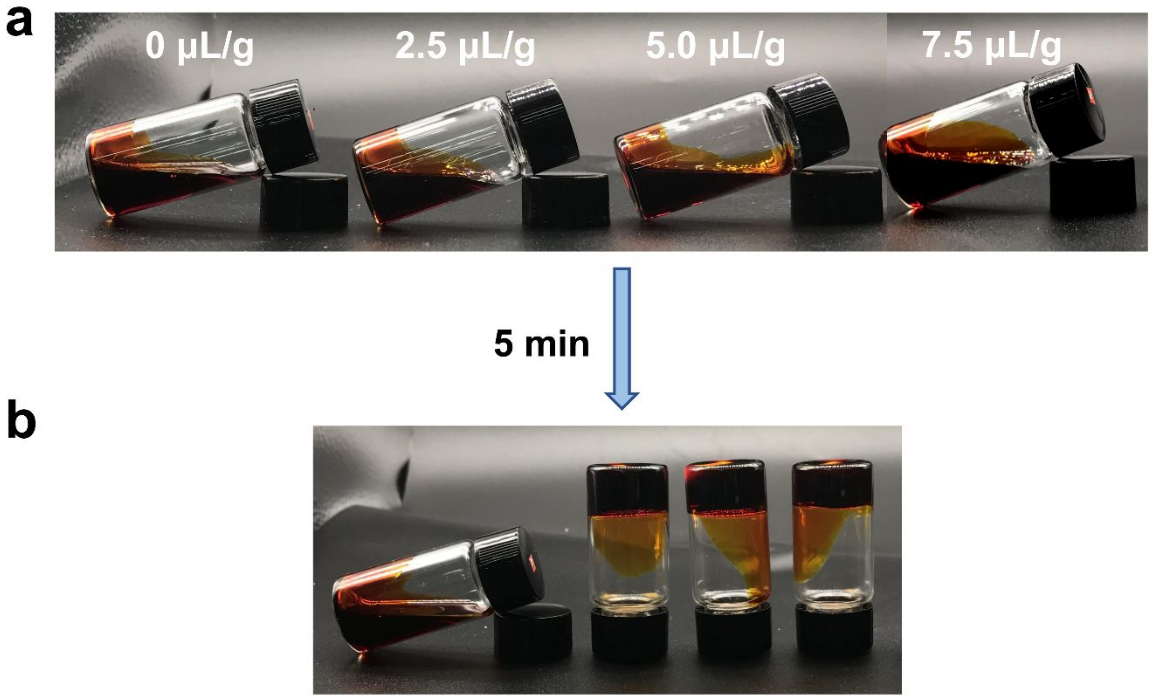

Figure S7. Digital photos of KNF ink mixed with different content of Db. After 5 min, the KNF ink mixed with different content of Db was gelled, all of which demonstrated that Db could form covalent alkane bonds at the negative ion site as the cross-linking agent.

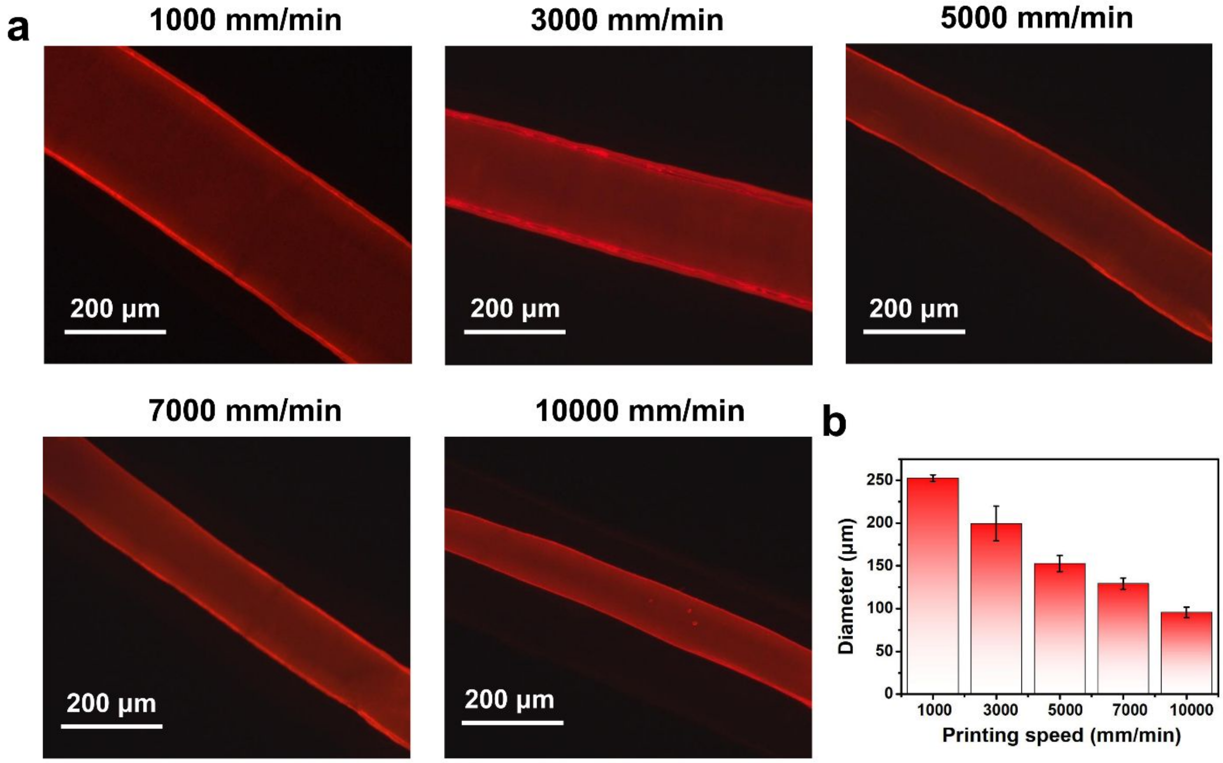

Figure S8. (a) Fluorescence micrographs and (b) histogram of the 3D printed KNF filaments. The diameter of filaments decreased with the increasing printing speed.

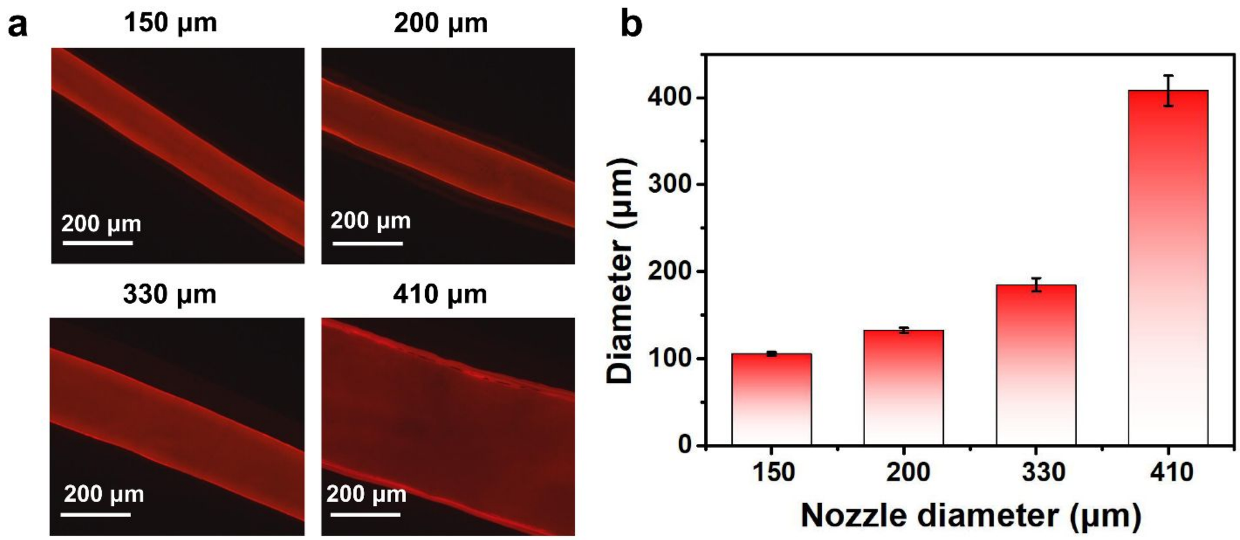

Figure S9. (a) Fluorescence micrographs and (b) histogram of the 3D printed KNF filaments. The diameter of filaments increased with the increasing nozzle diameter.

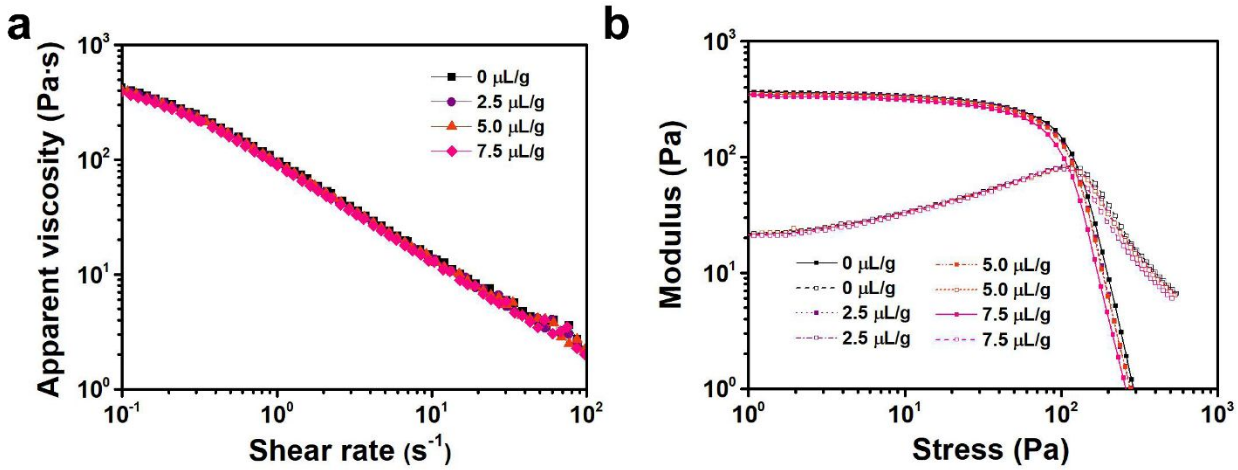

Figure S10. Rheological properties of the 2.5 wt% Carbopol 940/DMSO microgel matrix with different content of Db. (a) Log-log plot of apparent viscosity as a function of shear rate, showing an obvious shear thinning behavior where viscosity was dependent on the desired shear rate. (b) Log-log plot of dynamic stress sweep as a function of shear stress from 1 to 10^3 Pa at a constant frequency of 1 Hz, showing the similar storage and loss modulus was 350 Pa and 20 Pa respectively. All of which demonstrated that the addition of Db had almost little impact on the rheological properties of the matrix.

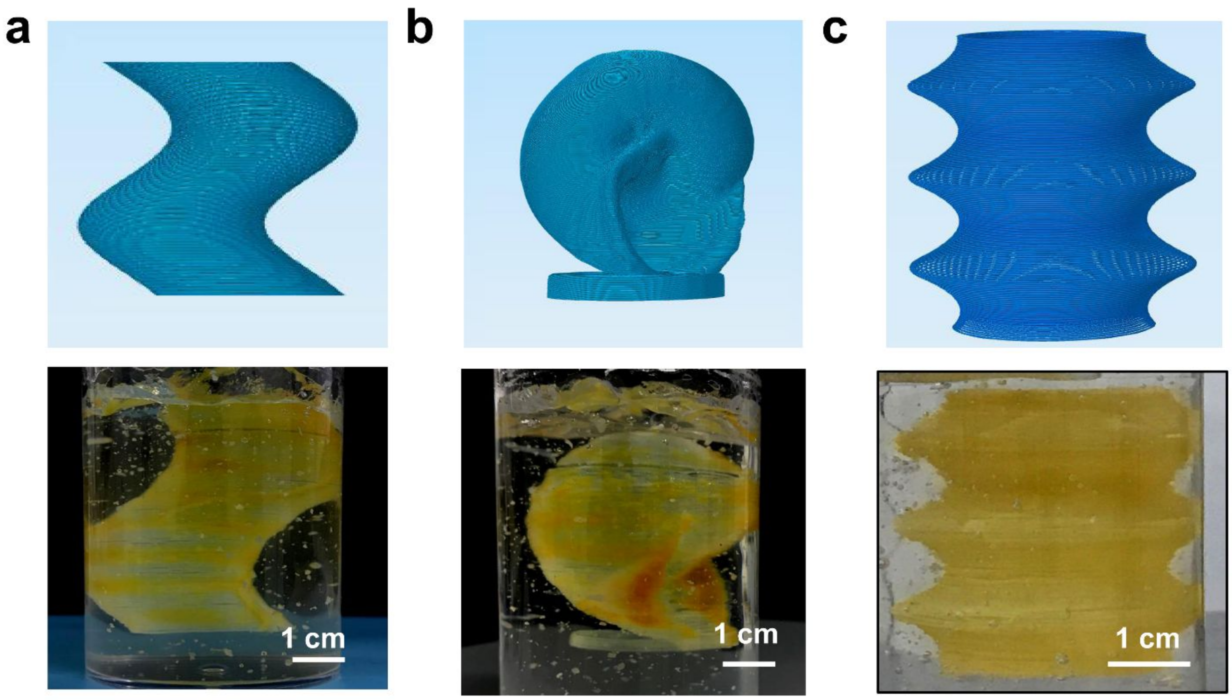

Figure S11. The as-designed models and printed 3D KNF gel with various shell structures. (a) tortuosity, (b) trumpet shell, (c) flexible tube, showing the outstanding shaping and programmability.

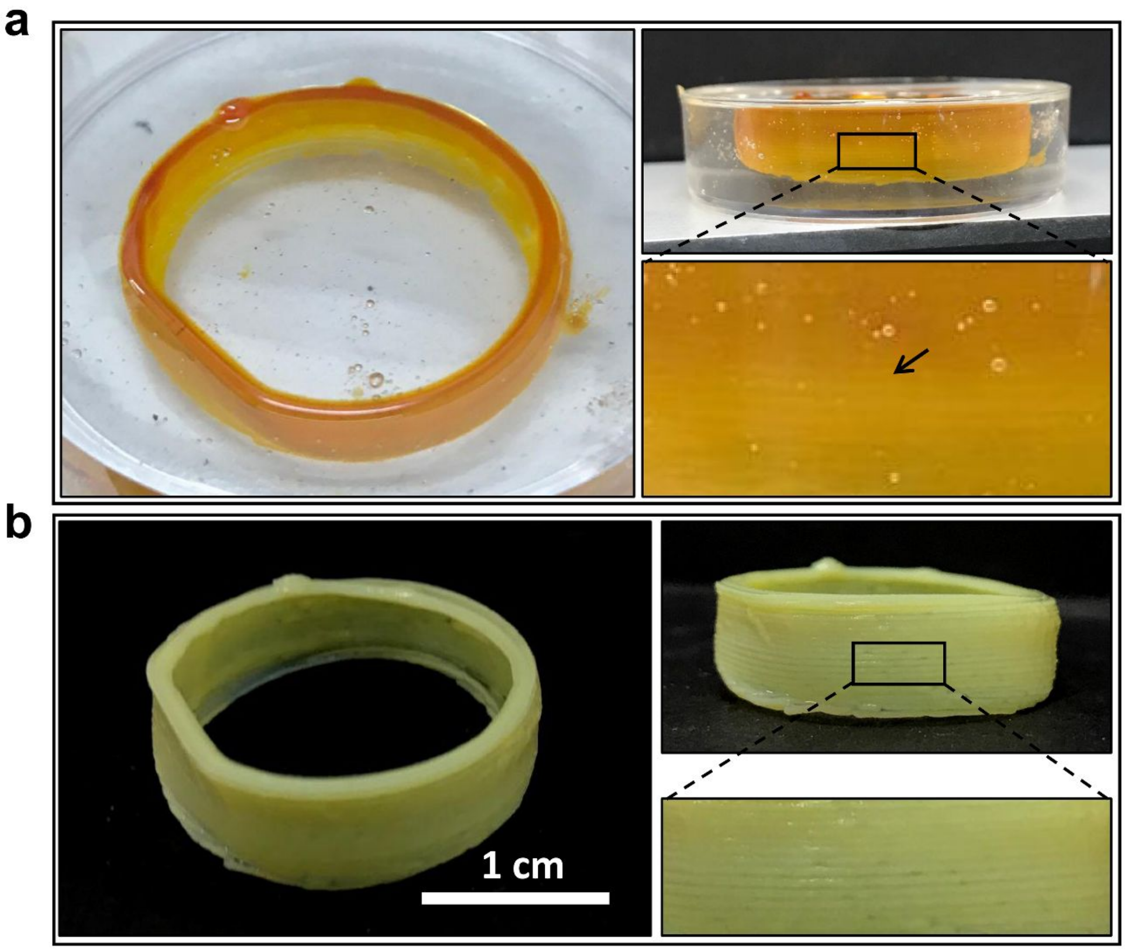

Figure S12. Digital photos of (a) the fresh 3D printed KNF architecture in the microgel matrix after the MSP process and (b) completely gelatinous 3D printed KNF architecture, both two exhibited almost interlayer adhesion.

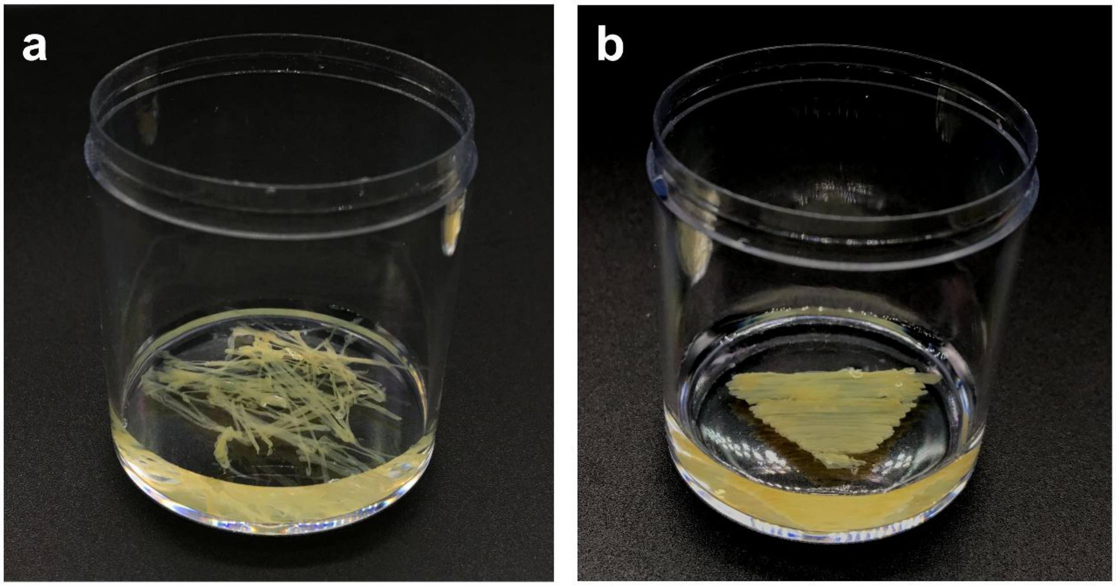

Figure S13. Digital photos of 3D printed KNF gel washed with water: the 3D printed KNF gel in (a) was prepared through 3D printing KNF inks in 2.5 wt% Carbopol 940/DMSO without Db, and the 3D printed KNF gel in (b) was prepared through 3D printing KNF inks in 2.5 wt% Carbopol 940/DMSO with Db of 5 μL g^{-1}. The structure of 3D printed KNF gel in (a) was broken while that in (b) was still maintain the shape, which demonstrated that the Db was essential to the structural integrity of 3D printed KNF aerogel.

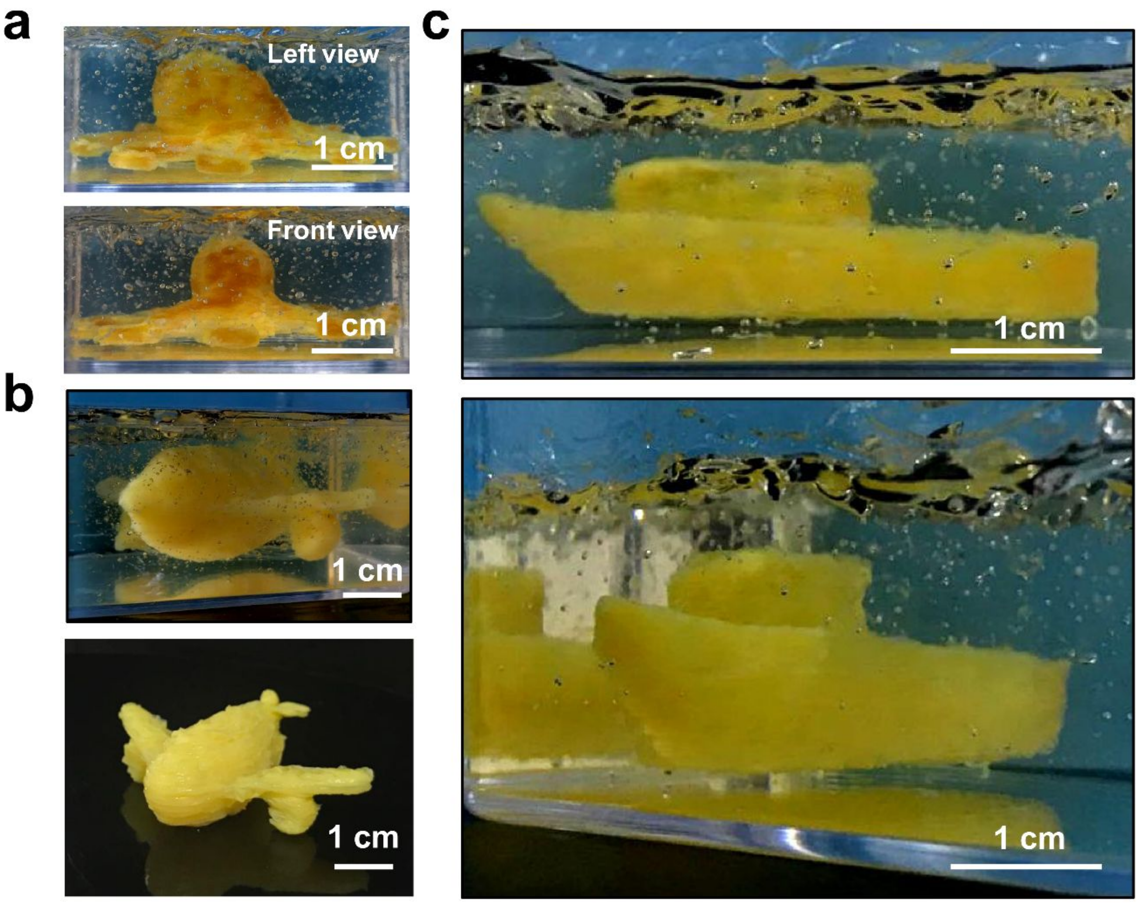

Figure S14. The as-designed and printed 3D KNF gel architectures with (a) octopus, (b) aircraft, (c) vessel. The architectures could be taken out from the matrix after the printing process had finished, showing superior interlayer adhesion.

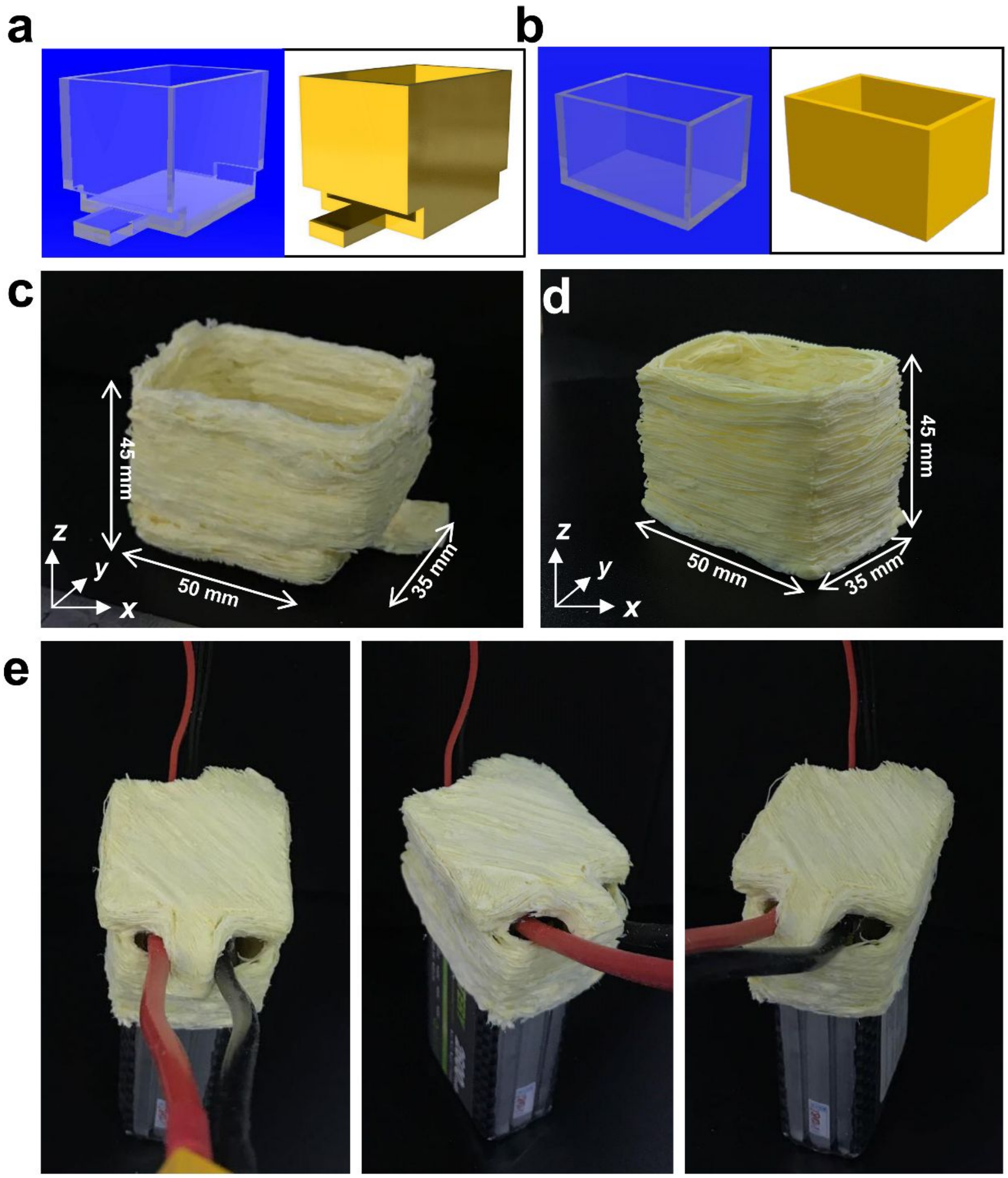

Figure S15. The (a-b) designed and (c-d) printed KAI-1 and KAI-2. The printed KNF filaments were deposited into an as-designed spatial stereoscopic structure in the layer-by-layer form. (e) The digital photos of D-LIPO coated with 3D-KAI-1 and 3D-KAI-2, which provided thermal

insulation protection under extremely low temperatures.

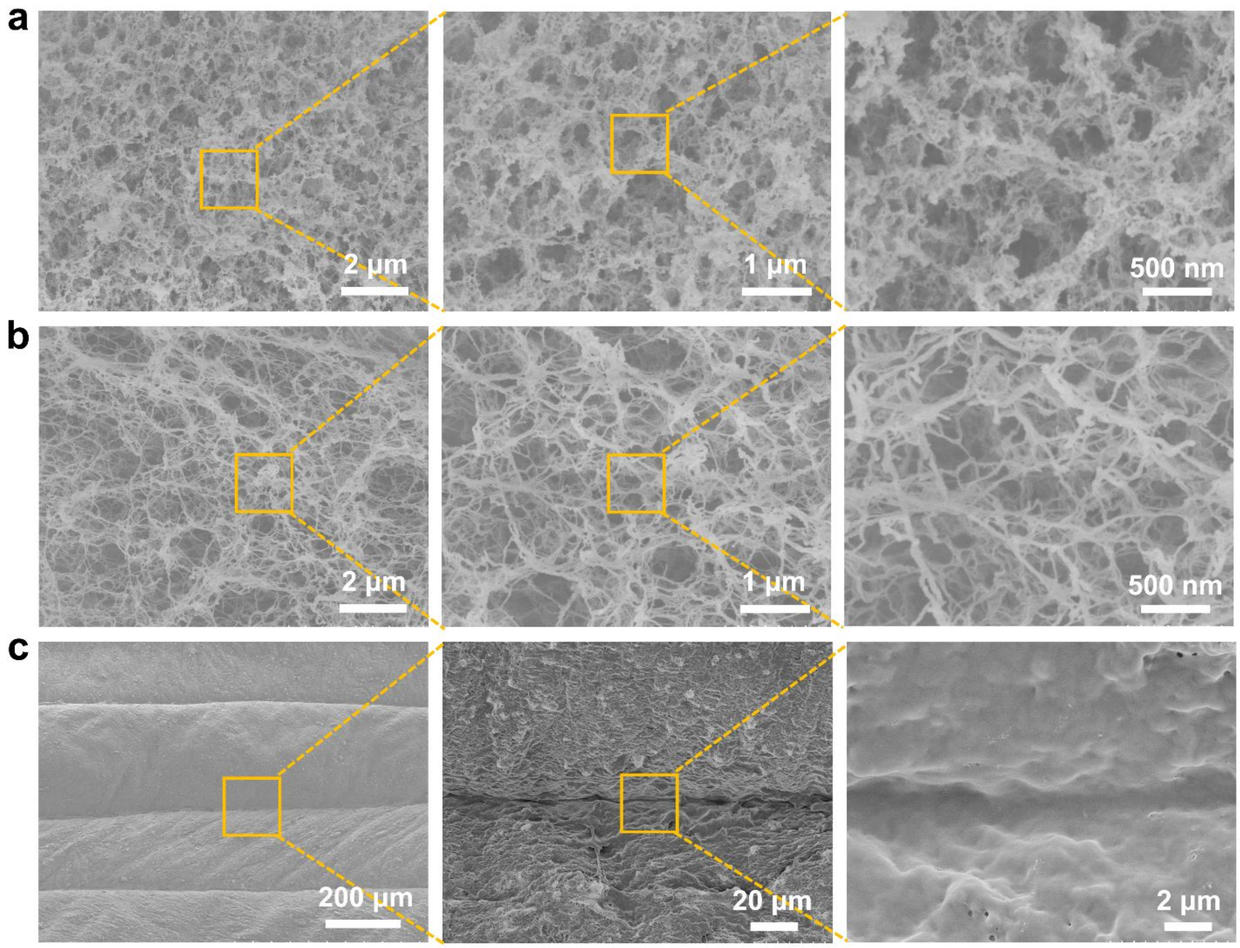

Figure S16. SEM images of cross-section view of (a) the 3D-KAI printed through 2.5 wt% Carbopol 940/DMSO microgel matrix with Db of 5 μL g^{-1} and (b) the reported KNF aerogel. (c) SEM images of the surface view of the 3D-KAI printed through 2.5 wt% Carbopol 940/DMSO with Db of 5 μL g^{-1}. SEM images in (a) and (b) demonstrated that an internal porous network consisting of denser entangled nanofibers interconnected with each other than reported KNF aerogel. The close-up micrograph of the surface in (c) exhibited a fine adhesion between adjacent

KNF filaments.

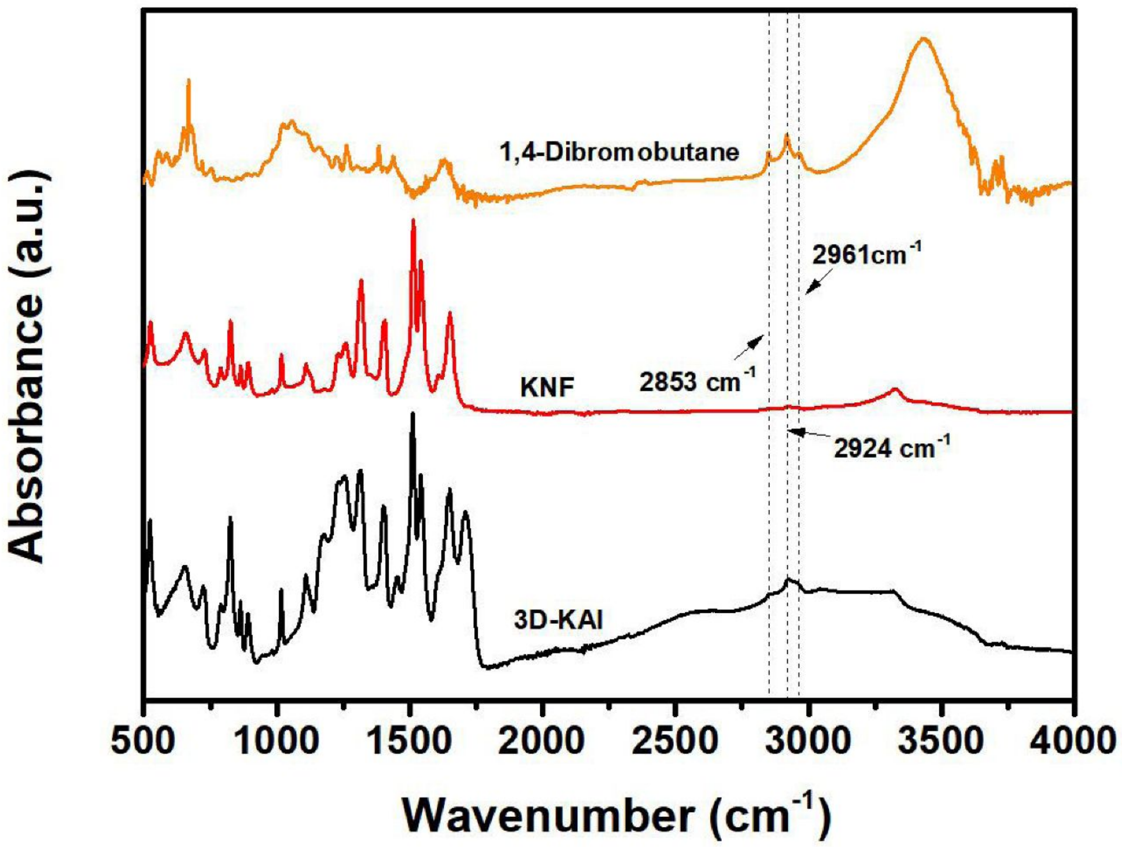

Figure S17. FTIR spectra of the KNF and 3D-KAI with Db content of 5 μL g^{-1}. FTIR spectra reveal that the Db was grafted on the KNF successfully due to the appearance of the characteristic peak of alkyl peak at 2853 cm^{-1}, 2924 cm^{-1} and 2961 cm^{-1}.

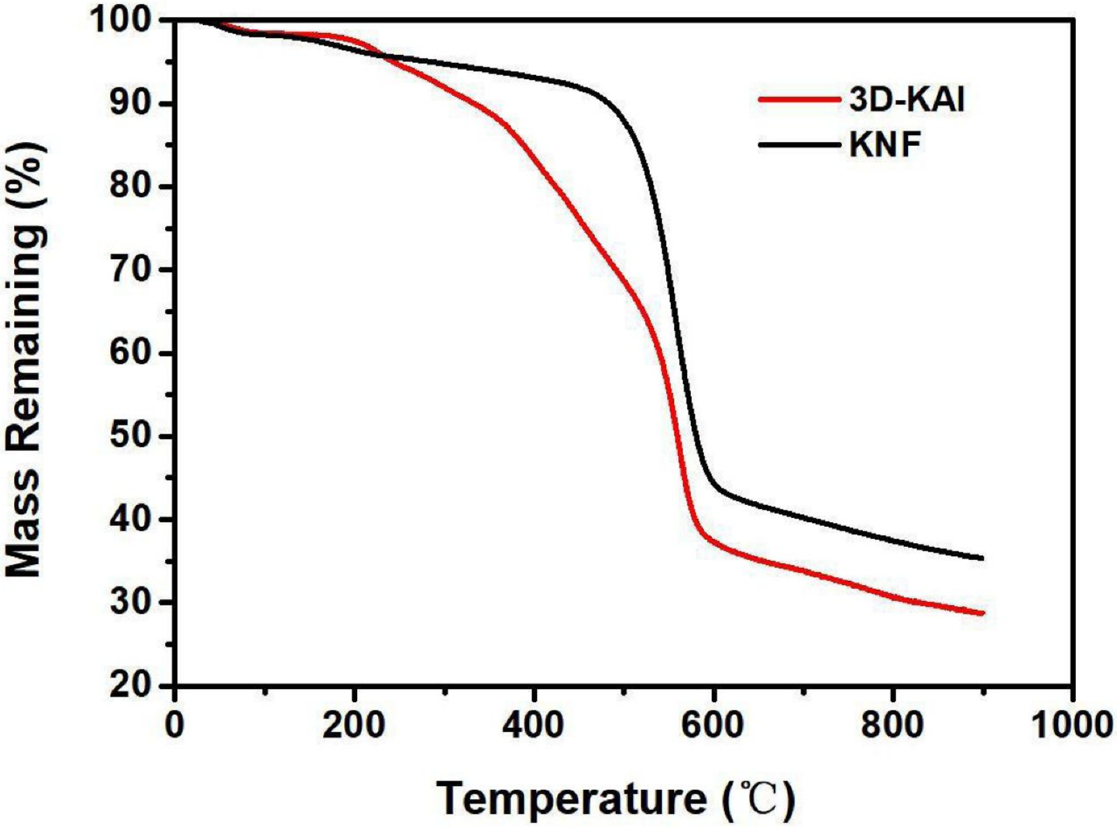

Figure S18. TGA curves for the KNF and 3D-KAI with Db content of 5 μL g^{-1}. The main decomposition process of 3D-KAI and KNF appeared at 508.4°C-580.9°C and 513.2°C-587.6°C respectively, indicating the introduction of alkyl groups had little effect on thermal stability.

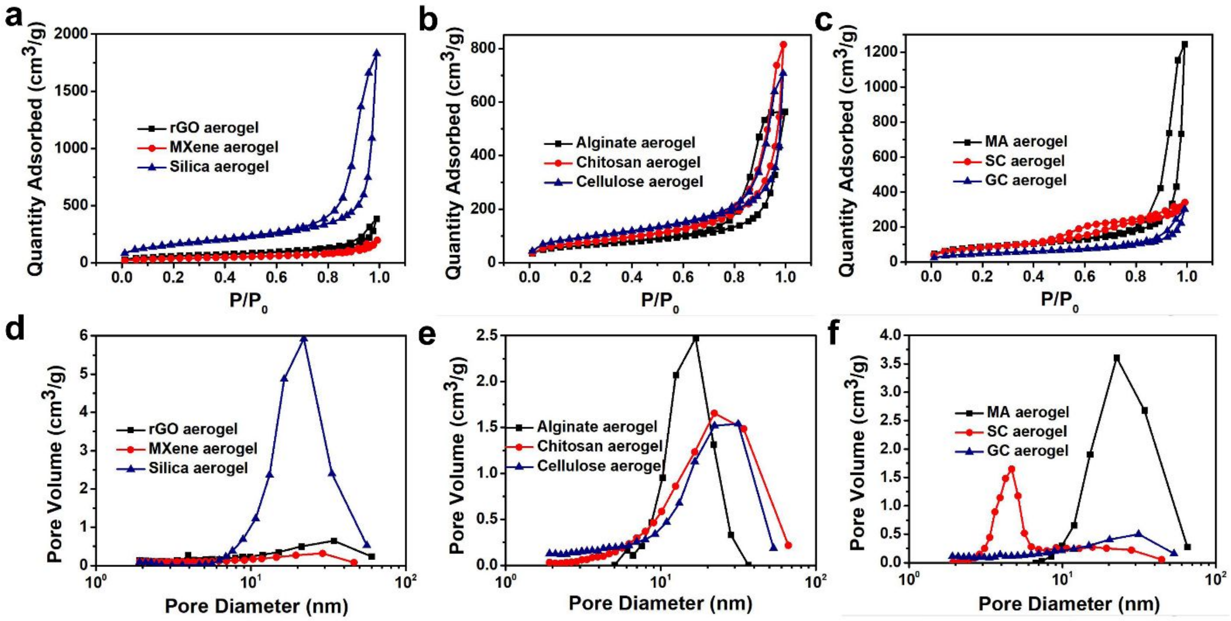

Figure S19. Nitrogen adsorption-desorption isotherms (a-c) and pore volume distribution (d-f) of the printed various organic, inorganic, and hybrid aerogels via MSP strategy.

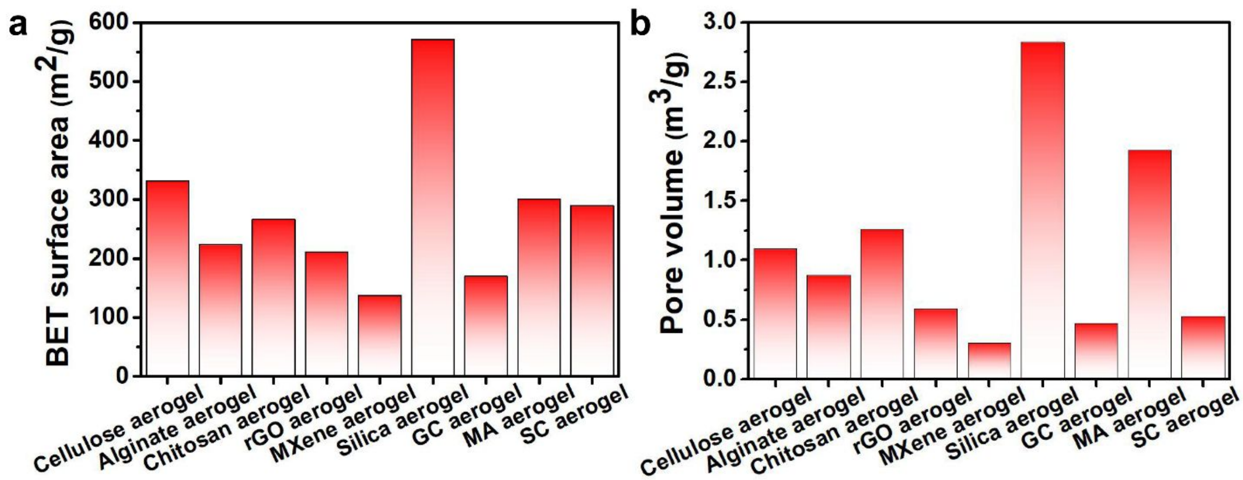

Figure S20. The (a) BET surface areas and (b) pore volumes of the printed various organic, inorganic, and hybrid aerogels via MSP strategy, demonstrating the universality of the MSP strategy for printing various aerogels.

Table S1. The comparison of rheological parameters of reported ink materials and the printing speeds to reported references via extruded-based DIW process.

Ink Material	η_a (Pa·s)	G' (Pa)	τ_y (Pa)	v_p (mm s^{-1})	Reference
Silk	1200	20000	—	1-10	Ref.[22]
Cellulose	1000	6000	350	10-20	Ref.[1]
Graphene	25-600	800-100000	30-650	—	Ref.[23]
Cellulose	40-150	5000	300-800	2	Ref.[2]
Kevlar	0.4-20	350-8000	4.38-180	16.7-167	This work
Cellulose	—	—	—	2	Ref.[3]
Cellulose	0.06	0.14	—	42	This work
Cellulose	50-250	—	—	2	Ref.[4]
Alginate	62.87	88	—	35	This work
Graphene	—	—	—	3.5	Ref.[5]
Chitosan	0.46	0.3	—	40	This work
Graphene	—	—	—	3	Ref.[6]
rGO	47.77	377	38	45	This work
Graphene	400	200000-40000	400-500	5	Ref.[7]
MXene	0.75	18.33	0.48	47	This work
Graphene	70-1000	4000-60000	100-1000	4-10	Ref.[8]
Silica	0.61	0.57	0.02	56	This work
Graphene	—	—	—	3	Ref.[9]
GC	16.17	134	12	52	This work
Graphene	150-300	70000-80000	1500-2000	4-20	Ref.[10]
MA	55.32	150	4.5	40	This work
Graphene	300-850	1500-40000	40-400	4	Ref.[11]
SC	0.39	0.3	—	40	This work
Graphene	—	—	—	10	Ref.[12]
Graphene	35-600	300-25000	30-1000	4-6	Ref.[13]
Graphene	400	15000	550	1-5	Ref.[14]
Graphene	100	5000-8000	150-800	4-6	Ref.[15]
Kevlar	25	8000	70	14	Ref.[16]
BN	1000-2000	70w-500w	2000-7000	1-10	Ref.[17]
Silica	10-400	60-10000	0.4-500	12-18.4	Ref.[18]
RF	100-200	6000	—	3	Ref.[19]
$g\text{-}C_3N_4$	400	10000	320	3-6	Ref.[20]
Silk	—	—	—	10	Ref.[21]

Benefiting from the assistance of the microgel matrix, the MS strategy broke the limitation of the requirements of rheological properties for DIW and improved the printing speed to some extent.

Movie S1. 3D printing process of Kevlar nanofiber ink via the MSP strategy.

Movie S2. 3D printing process of organic inks via the MSP strategy.

Movie S3. 3D printing process of inorganic inks via the MSP strategy.

Movie S4. 3D printing process of organic/inorganic hybrid ink via the MSP strategy.

References

(1) Siqueira, G.; Kokkinis, D.; Libanori, R.; Hausmann, M. K.; Gladman, A. S.; Neels, A.; Tingaut, P.; Zimmermann, T.; Lewis, J. A.; Studart, A. R. Cellulose Nanocrystal Inks for 3D Printing of Textured Cellular Architectures. *Adv. Funct. Mater.* **2017**, *27*, 1604619.

(2) Cao, D.; Xing, Y.; Tantratian, K.; Wang, X.; Ma, Y.; Mukhopadhyay, A.; Cheng, Z.; Zhang, Q.; Jiao, Y.; Chen, L.; Zhu, H. 3D Printed High-Performance Lithium Metal Microbatteries Enabled by Nanocellulose. *Adv. Mater.* **2019**, *31*, 1807313.

(3) Li, V. C.-F.; Dunn, C. K.; Zhang, Z.; Deng, Y.; Qi, H. J. Direct Ink Write (DIW) 3D Printed Cellulose Nanocrystal Aerogel Structures. *Scientific Reports* **2017**, *7*, 8018.

(4) Espinosa, E.; Filgueira, D.; Rodriguez, A.; Chinga-Carrasco, G. Nanocellulose-Based Inks-Effect of Alginate Content on the Water Absorption of 3D Printed Constructs. *Bioengineering* **2019**, *6*, *6030065*.

(5) An, B.; Ma, Y.; Li, W.; Su, M.; Li, F.; Song, Y. Three-Dimensional Multi-Recognition Flexible Wearable Sensor via Graphene Aerogel Printing. *Chem. Commun.* **2016**, *52*, 10948-10951.

(6) Yan, J.; Zhi, G.; Kong, D.; Wang, H.; Xu, T.; Zang, J.; Shen, W.; Xu, J.; Shi, Y.; Dai, S.; Li, X.; Wang, Y. 3D Printed rGO/CNT Microlattice Aerogel for a Dendrite-Free Sodium Metal Anode. *J. Mater. Chem. A* **2020**, *8*, 19843-19854.

(7) Fu, K.; Wang, Y.; Yan, C.; Yao, Y.; Chen, Y.; Dai, J.; Lacey, S.; Wang, Y.; Wan, J.; Li, T.; Wang, Z.; Xu, Y.; Hu, L. Graphene Oxide-Based Electrode Inks for 3D-Printed Lithium-Ion Batteries. *Adv. Mater.* **2016**, *28*, 2587-2594.

(8) Jiang, Y.; Xu, Z.; Huang, T.; Liu, Y.; Guo, F.; Xi, J.; Gao, W.; Gao, C. Direct 3D Printing of Ultralight Graphene Oxide Aerogel Microlattices. *Adv. Funct. Mater.* **2018**, *28*, 1707024.

(9) Yao, B.; Chandrasekaran, S.; Zhang, J.; Xiao, W.; Qian, F.; Zhu, C.; Duoss, E. B.; Spadaccini, C. M.; Worsley, M. A.; Li, Y. Efficient 3D Printed Pseudocapacitive Electrodes with Ultrahigh MnO_2 Loading. *Joule* **2019**, *3*, 459-470.

(10) Peng, M.; Wen, Z.; Xie, L.; Cheng, J.; Jia, Z.; Shi, D.; Zeng, H.; Zhao, B.; Liang, Z.; Li, T.; Jiang, L. 3D Printing of Ultralight Biomimetic Hierarchical Graphene Materials with Exceptional Stiffness and Resilience. *Adv. Mater.* **2019**, *31*, 1902930.

(11) Tang, X.; Zhou, H.; Cai, Z.; Cheng, D.; He, P.; Xie, P.; Zhang, D.; Fan, T. Generalized 3D Printing of Graphene-Based Mixed-Dimensional Hybrid Aerogels. *ACS Nano* **2018**, *12*, 3502-3511.

(12) Jose Moyano, J.; Garcia, I.; de Damborenea, J.; Perez-Coll, D.; Belmonte, M.; Miranzo, P.; Isabel Osendi, M. Remarkable Effects of an Electrodeposited Copper Skin on the Strength and the Electrical and Thermal Conductivities of Reduced Graphene Oxide-Printed Scaffolds. *ACS Appl. Mater. Interfaces* **2020**, *12*, 24209-24217.

(13) Guo, F.; Jiang, Y.; Xu, Z.; Xiao, Y.; Fang, B.; Liu, Y.; Gao, W.; Zhao, P.; Wang, H.; Gao, C. Highly Stretchable Carbon Aerogels. *Nat. Commun.* **2018**, *9*, 881.

(14) Yao, Y.; Fu, K. K.; Yan, C.; Dai, J.; Chen, Y.; Wang, Y.; Zhang, B.; Hitz, E.; Hu, L. Three-Dimensional Printable High-Temperature and High-Rate Heaters. *ACS Nano* **2016**, *10*, 5272-5279.

(15) Guo, F.; Zheng, X.; Liang, C.; Jiang, Y.; Xu, Z.; Jiao, Z.; Liu, Y.; Wang, H. T.; Sun, H.; Ma, L.; Gao, W.; Greiner, A.; Agarwal, S.; Gao, C. Millisecond Response of Shape Memory Polymer Nanocomposite Aerogel Powered by Stretchable Graphene Framework. *ACS Nano* **2019**, *13*, 5549-5558.

(16) Cheng, Q.; Liu, Y.; Lyu, J.; Lu, Q.; Zhang, X.; Song, W. 3D Printing-Directed Auxetic Kevlar Aerogel Architectures with Multiple Functionalization Options. *J. Mater. Chem. A* **2020**, *8*, 14243-14253.

(17) Liang, Z.; Pei, Y.; Chen, C.; Jiang, B.; Yao, Y.; Xie, H.; Jiao, M.; Chen, G.; Li, T.; Yang, B.; Hu, L. General, Vertical, Three-Dimensional Printing of Two-Dimensional Materials with Multiscale Alignment. *ACS Nano* **2019**, *13*, 12653-12661.

(18) Zhao, S.; Siqueira, G.; Drdova, S.; Norris, D.; Ubert, C.; Bonnin, A.; Galmarini, S.; Ganobjak, M.; Pan, Z.; Brunner, S.; Nyström, G.; Wang, J.; Koebel, M. M.; Malfait, W. J. Additive Manufacturing of Silica Aerogels. *Nature* **2020**, *584*, 387-392.

(19) Chandrasekaran, S.; Yao, B.; Liu, T.; Xiao, W.; Song, Y.; Qian, F.; Zhu, C.; Duoss, E. B.; Spadaccini, C. M.; Li, Y.; Worsley, M. A. Direct Ink Writing of Organic and Carbon Aerogels. *Mater. Horiz.* **2018**, *5*, 1166-1175.

(20) He, P.; Tang, X.; Chen, L.; Xie, P.; He, L.; Zhou, H.; Zhang, D.; Fan, T. Patterned Carbon Nitride-Based Hybrid Aerogel Membranes via 3D Printing for Broadband Solar Wastewater Remediation. *Adv. Funct. Mater.* **2018**, *28*, 1801121.

(21) Maleki, H.; Montes, S.; Hayati-Roodbari, N.; Putz, F.; Huesing, N. Compressible, Thermally Insulating, and Fire Retardant Aerogels through Self-Assembling Silk Fibroin Biopolymers Inside a Silica Structure-An Approach towards 3D Printing of Aerogels. *ACS Appl. Mater. Interfaces* **2018**, *10*, 22718-22730.

(22) Sommer, M. R.; Schaffner, M.; Carnelli, D.; Studart, A. R. 3D Printing of Hierarchical Silk Fibroin Structures. *ACS Appl. Mater. Interfaces* **2016**, *8*, 34677-34685.

(23) Zhu, C.; Han, T. Y.-J.; Duoss, E. B.; Golobic, A. M.; Kuntz, J. D.; Spadaccini, C. M.; Worsley, M. A. Highly Compressible 3D Periodic Graphene Aerogel Microlattices. *Nat. Commun.* **2015**, *6*, 6962.
